# Potential of extracellular vesicle-derived microRNAs as a platform for biomarker discovery in acute lymphoblastic leukemia

**DOI:** 10.1371/journal.pone.0352501

**Published:** 2026-06-24

**Authors:** Jeong-An Gim, Kunye Kwak, Yong Park, Byung Soo Kim, Ka-Won Kang

**Affiliations:** 1 Department of Medical Science, Soonchunhyang University, Asan, Republic of Korea; 2 Division of Hematology-Oncology, Department of Internal Medicine, Korea University College of Medicine, Seoul, Republic of Korea; European Institute of Oncology, ITALY

## Abstract

**Background:**

Extracellular vesicle (EV)-derived microRNAs (miRNAs) represent a promising platform for biomarker discovery in acute lymphoblastic leukemia (ALL). This study evaluated the biomarker potential of EV-derived miRNAs isolated from five ALL cell lines.

**Methods:**

Human ALL cell lines were cultured in EV-depleted fetal bovine serum. These included two parental cell lines, CCL-119 and CRL-3273, and three related cell lines previously characterized as exhibiting chemoresistance-associated phenotypes: CRL-2264 and CRL-2265, derived from CCL-119, and CRL-3274, derived from CRL-3273. EVs were isolated using a commercial size-exclusion chromatography-based method and characterized by nanoparticle tracking analysis, transmission electron microscopy, and immunoblotting for CD9, CD63, and CD81. Small RNA sequencing was subsequently performed. All data processing and visualization were conducted using R statistical software.

**Results:**

Across all samples, 2,656 EV-derived miRNAs were identified. Among these, three EV-derived miRNAs were prioritized based on consistent directional differences in this exploratory analysis: miR-1226-5p and miR-760 were downregulated, whereas miR-29b-3p was upregulated. To further assess their potential clinical relevance, we evaluated associations between survival and the expression of predicted target genes using the GSE5314 dataset. Higher expression of *AHI1*, a gene implicated in leukemogenesis and drug resistance and linked to downregulated miR-760 in our models, was associated with poor survival in patients with ALL.

**Conclusions:**

This exploratory study identified three EV-derived miRNAs, miR-1226-5p, miR-760, and miR-29b-3p, together with the related gene *AHI1*, as candidate biomarker leads in ALL cell line models. Further validation using patient-derived EVs, plasma samples, and subtype-aware clinical cohorts is required before clinical interpretation.

## Introduction

Acute lymphoblastic leukemia (ALL) is a malignancy of the lymphoid hematopoietic lineage characterized by the uncontrolled proliferation of immature lymphocytes. Survival outcomes in ALL have improved substantially with the incorporation of pediatric-inspired chemotherapy, allogeneic hematopoietic cell transplantation, tyrosine kinase inhibitors, bispecific antibodies, antibody-drug conjugates, and chimeric antigen receptor T-cell therapy [1–9]. Nevertheless, treatment responses and prognosis remain highly heterogeneous across patients, underscoring the need for biomarkers that can refine risk stratification and support therapeutic decision-making.

Genetic abnormalities play central roles in the pathogenesis and prognosis of ALL and have therefore been widely used as biomarkers [10]. However, these abnormalities may evolve during treatment [11], and their clinical implementation can be constrained by cost, turnaround time, technical complexity, and interpretability. In this context, extracellular vesicle (EV)-derived microRNAs (miRNAs) have emerged as promising biomarker candidates. MiRNAs regulate gene expression through post-transcriptional interactions with messenger RNAs (mRNAs) and may reflect dynamic changes in leukemic biology [12]. Moreover, encapsulation within EVs protects miRNAs from degradation in circulation, supporting their potential use in minimally invasive biomarker discovery [13].

In this study, we isolated EVs from ALL cell lines using a commercial size-exclusion chromatography-based method and evaluated EV-derived miRNAs as candidate biomarkers in ALL. Specifically, we aimed to identify EV-derived miRNAs associated with chemoresistance-related phenotypes in representative ALL cell line models and to explore their potential relevance to biomarker discovery. Accordingly, this cell line-based analysis was designed to nominate EV-miRNA candidates for future validation in clinically relevant samples.

## Materials and methods

### Cell culture

Human ALL cell lines were obtained from the American Type Culture Collection (ATCC; Manassas, VA, USA), comprising two parental cell lines (CCL-119 and CRL-3273) and three derivative cell lines previously described as exhibiting chemoresistance-associated phenotypes (CRL-2264 and CRL-2265, derived from CCL-119, and CRL-3274, derived from CRL-3273) (S1 File). These cells were cultured in EV-depleted fetal bovine serum according to the manufacturer’s instructions. EV-depleted fetal bovine serum was prepared by ultracentrifugation, in which standard fetal bovine serum was ultracentrifuged at 100,000 × g for 3 h. The supernatant was collected after removal of the pellet to reduce contamination by bovine EVs.

### EV isolation from cell culture supernatant

Conditioned medium from subconfluent cultures was harvested and sequentially centrifuged at 500 × g for 10 min, 5,000 × g for 30 min at 4 °C, and 10,000 × g for 30 min at 4 °C to remove detached cells, apoptotic bodies, and cellular debris. EVs were isolated using a size-exclusion chromatography-based method (EXo-i S5; Exopert, Seoul, Republic of Korea) with a two-layer column containing CL-6B Sepharose and Sephacryl S-200 High Resolution, according to the manufacturer’s instructions [14]. To obtain a more concentrated EV sample, the eluted fractions (10 and 11; 0.5 mL each, for a total volume of 1.0 mL) were concentrated using an Amicon® Ultra 100 kDa filter unit with a molecular weight cutoff of 100 kDa (Merck Millipore, Temecula, CA, USA).

### Nanoparticle tracking analysis and transmission electron microscopy

Nanoparticle tracking analysis (NTA) was performed to assess particle size distribution and concentration, and transmission electron microscopy (TEM) was used to evaluate EV morphology. NTA was conducted using a NanoSight NS300 instrument (Malvern Panalytical Ltd., Malvern, UK). The dynamic motion of EVs was recorded and analyzed using NTA software version 3.4 (Malvern Panalytical Ltd.). In accordance with current EV reporting recommendations [15], NTA-derived abundance values are reported as particle concentrations (particles/mL). NTA measurements were performed in at least three independent runs to assess particle size distribution and concentration.

TEM was performed using a Tecnai G2 F30 instrument (FEI, OR, USA) at 200 kV. For sample preparation, EV samples were mixed with 2% paraformaldehyde at a 1:1 ratio and incubated at room temperature for 15 min. The grid was then placed on 20 μL of the EV sample on Parafilm. After 30 min, the grid was washed three times with phosphate-buffered saline and fixed with 2.5% glutaraldehyde in phosphate-buffered saline. Following a 10-min incubation, the grid was washed five times with deionized water. Residual solution was removed by gentle blotting with filter paper, and the grid was allowed to dry completely.

### Protein extraction and western blotting

Western blotting was performed to assess expression of EV markers CD9, CD63, and CD81. Protein concentration was measured using a bicinchoninic acid protein assay kit (Pierce, Rockford, IL, USA). For each sample, 20 µg of protein was separated by 10% sodium dodecyl sulfate–polyacrylamide gel electrophoresis and transferred onto a polyvinylidene difluoride membrane with a pore size of 0.2 µm (Bio-Rad, Hercules, CA, USA). After blocking with 3% bovine serum albumin (w/v) in Tris-buffered saline containing 0.1% Tween® 20 detergent for 1 h, membranes were incubated overnight at 4 °C with mouse anti-CD9 (sc-13118, Santa Cruz Biotechnology), mouse anti-CD63 (sc-365604, Santa Cruz Biotechnology), or mouse anti-CD81 (sc-166028, Santa Cruz Biotechnology) monoclonal antibodies, each diluted 1:1000. Goat anti-mouse IgG and HRP antibodies (1:1000; CNG004–0005, Cell Nest for Science) were used as secondary antibodies. Antibody–antigen reactions were visualized using western enhanced chemiluminescence substrate (Bio-Rad). Images were acquired using the Amersham ImageQuant 800 western blot imaging system (Cytiva, Little Chalfont, UK).

### RNA isolation and expression profiling

Total RNA was isolated from each sample using the miRNeasy Serum/Plasma Kit (QIAGEN, Hilden, Germany). Each 30 µL sample was mixed with 1 mL of QIAzol lysis buffer and processed according to the manufacturer’s instructions. RNA quality and quantity were evaluated using the Agilent 2100 Bioanalyzer with the RNA Pico Kit and Small RNA Kit (Agilent Technologies, Santa Clara, CA, USA).

For small RNA sequencing (RNA-seq), a single representative sample from each cell line (CCL-119, CRL-3273, CRL-2264, CRL-2265, and CRL-3274) was sequenced. Libraries were prepared using the SMARTer smRNA-Seq Kit for Illumina (Takara Bio, Shiga, Japan) according to the manufacturer’s instructions. Sequencing was performed on an Illumina HiSeq 2500 platform (Illumina, San Diego, CA, USA) to generate 51 bp single-end reads. Raw sequencing output ranged from approximately 8.30 × 10^7^ to 9.49 × 10^7^ total reads per sample. FASTQ files generated by Macrogen (Seoul, Republic of Korea) were used for primary data analysis. Key input and sequencing quality-control metrics are summarized in S2 File, with additional RNA input and sequencing quality-control metrics provided in the Supporting Information (S3–S4 Files).

Because a single representative small RNA-seq library was analyzed per cell line, replicate-based differential expression modeling and multiple-testing correction were not applied. The analysis was therefore conducted as an exploratory candidate-screening analysis, with emphasis on effect size and consistent directionality across paired parental–derivative comparisons.

### Bioinformatic analysis of miRNA sequencing data

For each miRNA, read counts were normalized to the total number of reads in each sample. Normalized values were calculated as reads per million (RPM) using the following formula: miRNA counts/total counts in each sample × 1,000,000. After normalization, miRNAs with an RPM value of zero were excluded. The log10 (RPM + 1) value was used as the expression level. Because small RNA sequencing was performed once per cell line, differential expression analysis was considered exploratory. Candidate miRNAs were prioritized based on consistent directionality across paired parental–derivative comparisons and effect-size criteria. Nominal P-values were used only for ranking purposes.

### Network analysis for mRNA–miRNA interaction

The TargetScan database was used to predict miRNA target genes. Raw mRNA–miRNA interaction data were downloaded from the file “Nonconserved_Site_Context_Scores.txt” and imported using the “fread” function of the R package, “data.table.” Entries beginning with “hsa” in the miRNA column, denoting *Homo sapiens*, were selected for subsequent analysis.

### Exploratory use of the GSE5314 mRNA cohort for indirect clinical contextualization

Because publicly available ALL EV-miRNA datasets with survival information were unavailable, an independent mRNA expression cohort (GSE5314) was used only for indirect clinical contextualization at the level of predicted target genes. The dataset was obtained through the cBioPortal interface and analyzed using R software (version 4.3.0; R Foundation for Statistical Computing, Vienna, Austria). For each gene, samples were dichotomized at the median expression value into high (top 50%) and low (bottom 50%) expression groups. Samples with missing expression values for a given gene were excluded from the analysis of that gene. Survival curves were estimated using the Kaplan–Meier method and compared using the log-rank test implemented in the R packages “survival” and “survminer.” Multivariable survival analyses were not performed because the clinical covariates required for adjustment were unavailable or incompletely annotated in the accessed dataset or through the portal interface.

### Ethics statement

This study used only established, de-identified human cell lines obtained from ATCC. No human participants were involved in this study, and no identifiable private information or biospecimens were used. Under the U.S. Common Rule (45 CFR 46.102), this work does not constitute human subjects research; therefore, Institutional Review Board review and informed consent were not required.

## Results

### Characterization of EVs derived from five ALL cell lines

**Fig 1:**
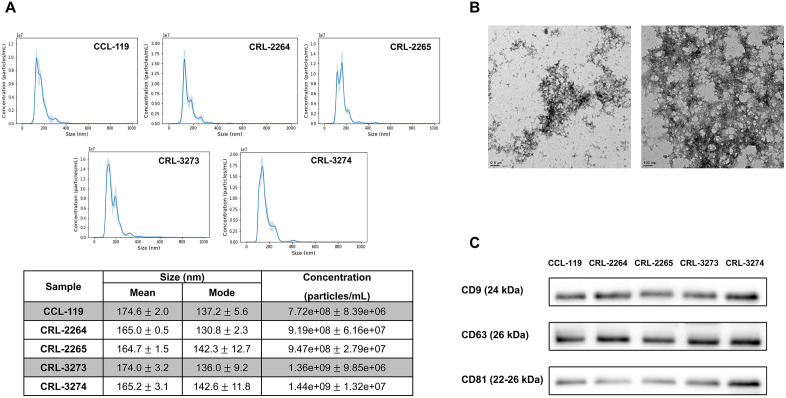
Characterization of extracellular vesicle (EV)-enriched preparations isolated from five acute lymphoblastic leukemia cell lines. (A) Particle size distribution and concentration measured by nanoparticle tracking analysis (NTA); values are shown as mean ± SD from at least three NTA runs per sample. (B) Transmission electron microscopy images showing vesicular morphology. (C) Immunoblotting for CD9, CD63, and CD81. Raw blot images are provided in S5 File.

The particle size of EVs derived from the ALL cell lines was 174.6 ± 2.0 nm for CCL-119, 165.0 ± 0.5 nm for CRL-2264, 164.7 ± 1.5 nm for CRL-2265, 174.0 ± 3.2 nm for CRL-3273, and 165.2 ± 3.1 nm for CRL-3274 (**Fig 1A**). Particle concentration in each sample was quantified using NTA (**Fig 1A**). TEM imaging showed EVs as cup-shaped vesicles smaller than 200 nm (**Fig 1B**). These findings supported the presence of EV-enriched preparations in the isolated samples. Western blotting confirmed the presence of the EV-associated markers CD9, CD63, and CD81 (**Fig 1C and** **S5 File**).

### Profiling miRNAs of EVs derived from five ALL cell lines

A total of 2,656 EV-derived miRNAs were identified across the five ALL cell lines. Among these, 482 miRNAs exhibited RPM values >0 in at least one of the five samples. These miRNAs were used to explore differences in miRNA expression patterns between the parental cell lines, CCL-119 and CRL-3273, and the cell lines previously described as exhibiting chemoresistance-associated phenotypes, namely CRL-2264, CRL-2265, and CRL-3274.

**Fig 2:**
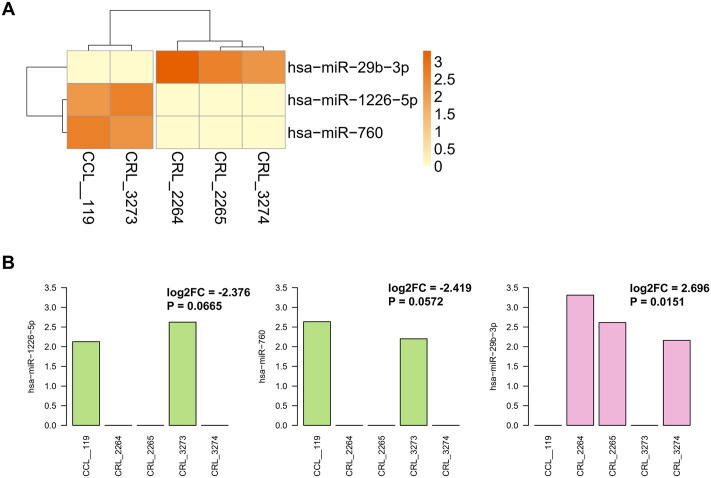
Exploratory prioritization of extracellular vesicle (EV)-derived miRNA candidates in chemoresistance-associated derivative acute lymphoblastic leukemia (ALL) cell lines. (A) Heat map of three EV-derived miRNAs (miR-1226-5p, miR-760, miR-29b-3p) across CCL-119, CRL-3273, CRL-2264, CRL-2265, and CRL-3274. (B) Bar plots showing lower expression of miR-1226-5p and miR-760, and higher expression of miR-29b-3p in cell lines previously described as exhibiting chemoresistance-associated phenotypes (CRL-2264, CRL-2265, and CRL-3274) relative to their matched parental cell lines (CCL-119 and CRL-3273). Note: Because a single representative small RNA-seq library was analyzed per cell line, these data should be interpreted as exploratory candidate-screening results. Nominal P-values are provided only for descriptive ranking, and replicate-based differential expression testing was not performed.

To screen for downregulated miRNAs in the derivative cell lines, two miRNAs were selected based on the following criteria: the absolute values of log10(RPM + 1) + 2 for each derivative cell line were greater than those of the corresponding parental cell line (CRL-2264 > CCL-119, CRL-2265 > CCL-119, and CRL-3274 > CRL-3273). For upregulated miRNA screening, one miRNA was selected using the following criteria: the absolute values of log10(RPM + 1) − 2 for each derivative cell line were less than those of the corresponding parental cell line (CRL-2264 < CCL-119, CRL-2265 < CCL-119, and CRL-3274 < CRL-3273). Three candidate EV-derived miRNAs showing consistent directional differences across paired models (miR-1226-5p, miR-760, and miR-29b-3p) were visualized as heatmaps across all cell lines (**Fig 2A**). The expression levels of miR-1226-5p and miR-760 were downregulated, whereas that of miR-29b-3p was upregulated (**Fig 2B**). Among the three candidates, miR-29b-3p met the nominal significance threshold in this exploratory screen (P < 0.05), whereas miR-1226-5p and miR-760 did not; however, these miRNAs were retained as screening candidates based on their consistent absence–presence pattern across models.

### Prioritization of EV-derived miRNA candidates and predicted miRNA–mRNA interactions in derivative cell lines with chemoresistance-associated phenotypes

A miRNA–mRNA network analysis was conducted to explore genes associated with the three EV-derived miRNAs identified in this study (miR-1226-5p, miR-760, and miR-29b-3p). Survival associations of predicted target genes were then evaluated to provide indirect clinical context for the prioritized EV-derived miRNAs.

**Fig 3:**
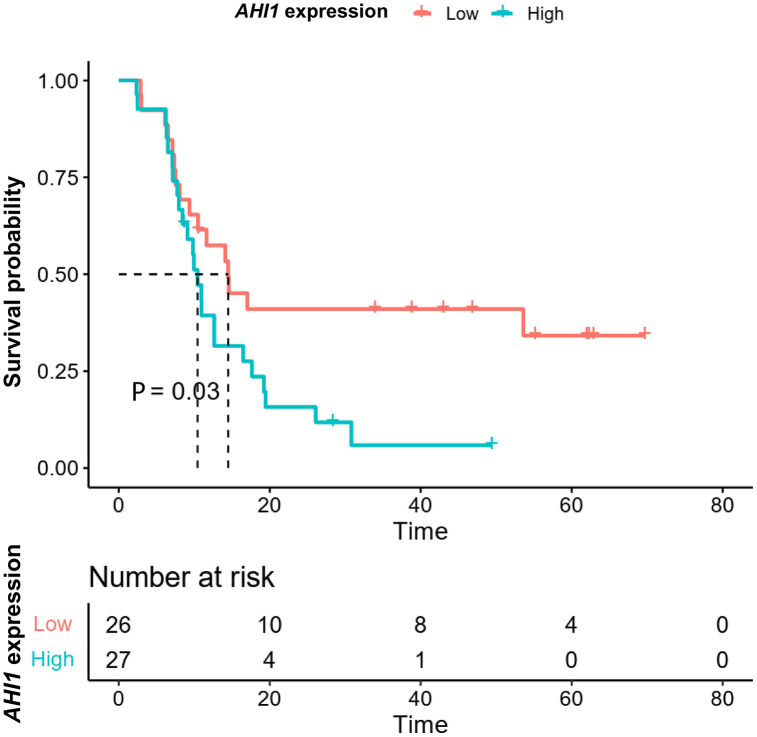
Kaplan–Meier survival curve according to Abelson helper integration site 1 (*AHI1)* expression in acute lymphoblastic leukemia (ALL). *AHI1* was selected for survival analysis as a high-confidence predicted target of miR-760 in TargetScan (context++ score = −0.466; percentile = 98; 3′UTR site: 308–315). High *AHI1* expression is associated with poorer survival in patients with ALL (log-rank P = 0.03). Note: The miR-760–*AHI1* relationship shown is based on TargetScan prediction and was not experimentally validated in the present study. Survival analysis was performed solely to provide indirect clinical contextualization at the predicted target gene level. Kaplan–Meier curves were compared using the log-rank test.

Among the 38,497,659 miRNA–mRNA interactions in the TargetScan database, 14,337,320 interactions corresponding to *H. sapiens* were selected. Genes associated with the three selected EV-derived miRNAs (miR-1226-5p, miR-760, and miR-29b-3p) were then filtered using a context++ score > 90. The context++ score ranges from 0 to 100, with a higher score indicating a greater likelihood of miRNA–mRNA targeting [16]. Using this approach, 75 mRNAs were identified for miR-1226-5p, 101 for miR-760, and 33 for miR-29b-3p; the complete target gene list is provided in S6 File. Next, to provide indirect clinical context for the prioritized predicted target genes, we analyzed the GSE5314 gene expression dataset. We first divided the samples into two groups based on the median expression level of each gene: “high” if the expression was above the median and “low” if it was below the median. Samples with missing expression values for a given gene were excluded from the analysis. In this analysis, higher expression of the gene encoding Abelson helper integration site 1 (*AHI1*), linked to downregulated miR-760, was associated with poorer survival in patients with ALL (**Fig 3**).

## Discussion

This study identified three EV-derived miRNAs, miR-1226-5p, miR-760, and miR-29b-3p, together with the related gene *AHI1*, as exploratory candidate biomarkers potentially linked to chemoresistance-related biology and poor outcomes in ALL. These findings support the potential utility of EV-derived miRNAs as a platform for biomarker discovery in ALL.

MiRNAs have been investigated for diagnosis, classification, prognosis, and drug resistance in ALL [17–19]. Previous studies have reported that downregulation of miR-128b [20] and overexpression of miR-17 [21] contribute to glucocorticoid resistance, whereas downregulation of miR-217 has been linked to tyrosine kinase inhibitor (TKI) resistance [22]. Chemoresistance has been linked to multiple miRNAs, including the downregulation of sol-miR-23 [23] and upregulation of miR-125b, miR-99a, and miR-100 [24]. In the present study, we identified downregulated miR-1226-5p and miR-760 and upregulated miR-29b-3p in cell lines previously described as exhibiting chemoresistance-associated phenotypes. Downregulation of miR-1226 has been reported to correlate with chemoresistant leukemia stem cells [25]. In contrast, changes in miR-760 and miR-29b-3p expression and their association with ALL have not been investigated. Previous studies have linked downregulated miR-760 expression to chemoresistance in other solid malignancies [26,27].

The finding regarding miR-29b-3p warrants particular caution. Across several malignancies, miR-29b-3p has frequently been reported to exhibit tumor-suppressive or chemosensitivity-related roles [28,29]. Nevertheless, direct evidence linking miR-29b-3p to chemosensitivity in ALL remains limited. Studies in ALL have primarily described altered miR-29b-3p expression and biological relevance, including inhibition of malignant behavior in T-cell ALL, suppression of ALL cell proliferation, and promotion of apoptosis through EV-mediated delivery, rather than a direct effect on chemosensitivity [30,31]. Moreover, our analysis focused on EV-derived miR-29b-3p rather than intracellular miR-29b-3p. Because miRNAs can be selectively sorted into EVs in a cell-state- and context-dependent manner, EV-miRNA abundance may not directly mirror intracellular activity [32]. Therefore, the upregulation of EV-derived miR-29b-3p observed in our models may reflect context-dependent EV-associated biology rather than a uniform functional effect. This result should be interpreted as an exploratory biomarker-associated observation. The present data do not establish a direct mechanistic role for EV-derived miR-29b-3p in chemoresistance, and further mechanistic studies and validation in clinically relevant samples are needed to clarify its role in ALL.

*AHI1* is a protein-coding gene implicated in the development of certain types of human leukemia through cooperation with oncogenes, such as v-abl and c-myc, as well as tumor suppressor genes [33]. *AHI1* is highly expressed in chemoresistant leukemia stem cells in chronic myeloid leukemia [34] and Philadelphia chromosome-positive human leukemias [35], and has been associated with poor prognosis in a subset of T-cell ALL [36]. Mechanistically, *AHI1* mediates the formation of a protein complex containing BCR-ABL and JAK2, which contributes to BCR-ABL transforming activity and TKI resistance [34,37,38]. In addition, suppression of *AHI1* has been shown to increase TKI sensitivity, even during blast crisis in patients with TKI-resistant chronic myeloid leukemia [39]. In our analysis, *AHI1* emerged as a hypothesis-generating predicted target within the miR-760-associated network detected in ALL cell lines previously described as exhibiting chemoresistance-associated phenotypes. In an independent publicly available dataset, higher *AHI1* expression was associated with poorer survival. These findings further support the potential utility of EV-derived miRNAs as biomarkers for ALL.

Recent studies have increasingly highlighted the translational relevance of EV-associated nucleic acids, including miRNAs, as minimally invasive cancer biomarkers [40–42]. EV cargo is protected during circulation and reflects the biologic features of the cells of origin, making EV-derived miRNAs relevant to diagnostic, prognostic, and treatment-monitoring applications in liquid biopsy approaches. EVs also function as mediators of tumor progression, intercellular communication, immune modulation, and therapy resistance, extending the potential biological relevance of EV-derived miRNA signatures beyond biomarker detection alone. In hematologic malignancies, emerging evidence suggests that EV-mediated signaling contributes to leukemogenesis and disease progression, with potential diagnostic, prognostic, and therapeutic implications [43]. Within this context, the EV-derived miRNAs identified in this study may represent candidate biomarkers associated with poor outcomes and potential components of EV-mediated leukemic communication networks, although these implications warrant further mechanistic and clinical validation.

This study has some limitations. First, the experiments were performed using established ALL cell line models, without validation in patient-derived EVs or plasma samples. Accordingly, these findings should not be interpreted as evidence of clinical biomarker validity, and the translational relevance and clinical utility of the identified EV-derived miRNAs remain uncertain. Second, the selected EV-derived miRNAs were identified through exploratory analyses in a limited number of representative cell line models. Third, because no publicly available miRNA datasets containing chemoresistance or survival information in ALL were available at the time of analysis, direct clinical validation of the selected EV-derived miRNAs could not be performed. The GSE5314 dataset was used solely to explore the clinical relevance of predicted target genes and does not provide direct validation of EV-derived miRNAs in patients with ALL. Accordingly, the GSE5314 analysis should be interpreted as an indirect, hypothesis-generating clinical context for the predicted miR-760–*AHI1* relationship. Multivariable survival analyses were also not performed because the key clinical covariates required for adjustment were unavailable or incompletely annotated in the accessed dataset or portal. Fourth, chemotherapy resistance was not experimentally validated in the cell lines used in this study. Therefore, these models should be interpreted as cell lines previously described as exhibiting chemoresistance-associated phenotypes, based on prior characterization. Fifth, the cell lines analyzed in this study included both T-cell and B-cell ALL models, with the B-lineage models representing a distinct molecular context [44–47]. Importantly, heterogeneity in ALL exists not only between B-ALL and T-ALL, but also within each lineage, where molecularly defined subgroups can differ substantially in pathogenesis, prognosis, and drug-response biology [48,49]. Therefore, the EV-miRNA candidates identified in this study should not be generalized across all ALL contexts. In addition, EV-associated signals in ALL may reflect context-dependent leukemia biology and microenvironment-related processes, further supporting cautious interpretation of findings derived from a mixed-model panel [50]. Validation in subtype-aware cohorts is necessary to determine whether these signals reflect lineage-level biology, chemoresistance-associated biology, or model-specific EV-miRNA sorting. Sixth, EV characterization did not include a comprehensive purity-related assessment or systematic negative-marker evaluation recommended in the current Minimal Information for Studies of Extracellular Vesicles guidance. Although EV characterization was performed using approaches commonly applied when the experiments were conducted, including NTA, TEM, and immunoblotting for CD9, CD63, and CD81, more comprehensive purity-related assessment and systematic negative-marker evaluation were not performed. Therefore, the findings should be interpreted as arising from EV-enriched preparations. Nevertheless, compared with prior studies that identified miRNAs associated with chemoresistance using patient samples, this study provides exploratory evidence for EV-derived miRNAs associated with chemoresistance-related phenotypes in established ALL cell line models. Additionally, we leveraged publicly available datasets to identify a gene associated with poor outcomes that was linked to EV-derived miRNAs identified in ALL cell line models previously described as exhibiting chemoresistance-associated phenotypes.

In conclusion, this study identified three EV-derived miRNAs (miR-1226-5p, miR-760, and miR-29b-3p) and the related gene *AHI1* as exploratory candidate biomarkers in ALL cell line models. EV-derived miRNAs may serve as a useful platform for biomarker discovery in ALL; however, validation in patient-derived EVs or plasma samples is required to confirm their clinical significance.

## Supporting information

S1 File Characteristics of ALL cell lines used in this study. This table summarizes the ATCC numbers, cell line names, lineage/disease models, parental or derivative status, paired comparators, previously described phenotypes or key characteristics, and sources of the ALL cell lines used in this study.
(XLSX)

S2 File Sample-level summary of input RNA and small RNA-sequencing quality metrics for EV samples from five ALL cell lines. This table summarizes total read bases, raw reads, GC content, Q20 and Q30 values, trimmed reads, remaining reads after rRNA removal, and the number of known miRNAs detected per sample.
(XLSX)

S3 File Original sample QC report. This file provides the original RNA sample quality-control report, including RNA concentration, final volume, total amount, QC result, and electropherogram-based quality assessment for the five samples.
(PDF)

S4 File *Homo sapiens* miRNA sequencing report. This file provides the small RNA-sequencing workflow, data production summary, preprocessing statistics, rRNA removal results, read length distribution, miRNA composition, mature miRNA quantification, known/novel miRNA prediction, and differentially expressed miRNA analysis results.
(PDF)

S5 File Raw Western blot images. This file contains the raw Western blot and corresponding membrane images for EV marker proteins CD9, CD63, and CD81 in CCL-119, CRL-2264, CRL-2265, CRL-3273, and CRL-3274 samples.
(PDF)

S6 File Predicted target genes associated with EV-derived miRNAs from chemoresistant ALL cell lines. This table lists EV-derived miRNAs, predicted target genes, fold changes, and P values, including predicted targets associated with miR-1226-5p, miR-760, and miR-29b-3p.
(XLSX)
