# Supplementary material for: Potential of extracellular vesicle-derived microRNAs as a platform for biomarker discovery in acute lymphoblastic leukemia
Source: PLoS One. 2026 Jun 24;21(6):e0352501. doi: 10.1371/journal.pone.0352501 (PMC13293457; doi:10.1371/journal.pone.0352501)
Supplement: S3 File — This file provides the original RNA sample quality-control report, including RNA concentration, final volume, total amount, QC result, and electropherogram-based quality assessment for the five samples. (PDF) [file pone.0352501.s003.pdf]

macrogen

Original Sample QC

General Information

Order Number	HN00163911	Name of Customer	강가원	Date of Order	2021-12-16

Final QC Result of DNA sample(s)					
Arrival Date	Experiment Date	Sample count	Pass	Fail	Hold
N/A	N/A	N/A	N/A	N/A	N/A

Final QC Result of RNA sample(s)					
Arrival Date	Experiment Date	Sample count	Pass	Fail	Hold
2021-12-16	2021-12-23	5	0	0	5

The QC criteria refer to the specification requirements of a single run. In any cases, we may encounter the shortage of sample volume or amount due to various reasons such as a library construction failure. In these cases a request of an additional sample will be inevitable.
Therefore, we recommend double the amount to be supplied at first place to minimize any delay of the whole procedure.

*** Pass : Proceed with the library construction.**

*** Fail : Further processes are on hold until the replacement samples received.**
We do not recommend in proceeding further steps until a specific instruction was given from the client.

*** Hold : A specific instruction should be given by the client for further processing as the QC pattern may be triggered by the sample nature.**
Macrogen does not proceed the next step until we have received your permission.

As 3 ul was taken from the sample for sample (library) QC purposes, the indicated volume represents 3ul less than the total volume received.

QC Result of RNA

Arrival Date	2021-12-16	Experiment Date	2021-12-23	Tested by	KJB
Comment					

#	Sample Name	Conc. (ng/ul)	Final Volume (ul)	Total Amount (ug)	Result*	
1	4 - 1	2.006	15	0.03	Hold	Need to confirm
2	4 - 2	1.418	15	0.021	Hold	Need to confirm
3	4 - 3	0.955	15	0.014	Hold	Need to confirm
4	4 - 4	1.905	15	0.029	Hold	Need to confirm
5	4 - 5	3.357	15	0.05	Hold	Need to confirm

Experiment Condition	Bioanalyzer RNA Pico 6000 chip

이미지 확대하기 =>1:4 - 1

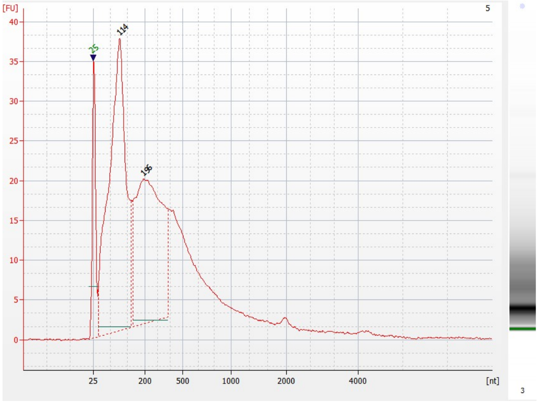

이미지 확대하기 =>2:4 - 2

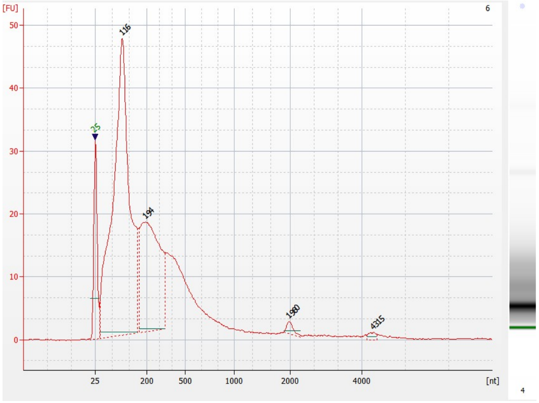

이미지 확대하기 =>3:4 - 3

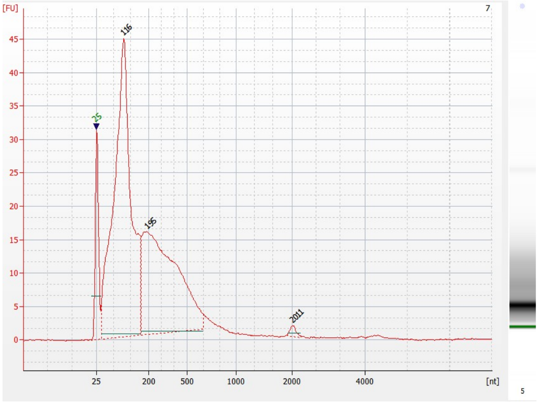

이미지 확대하기 =>4:4 - 4

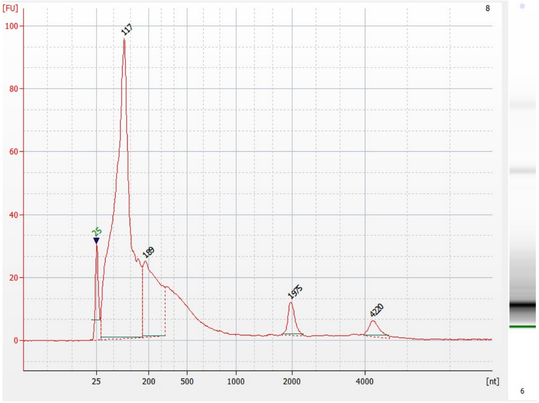

이미지 확대하기 =>5:4 - 5

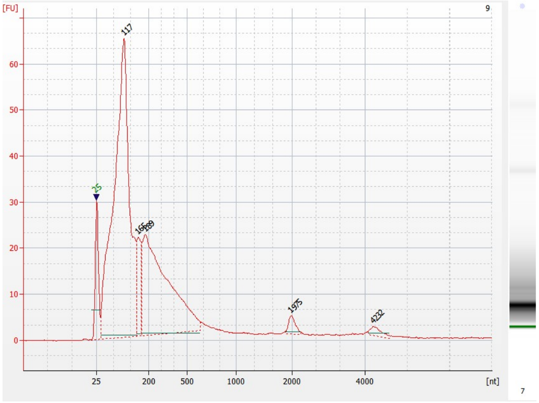

Experiment Condition	TapeStation HS RNA Screen Tape

이미지 확대하기 =>1:4 - 1

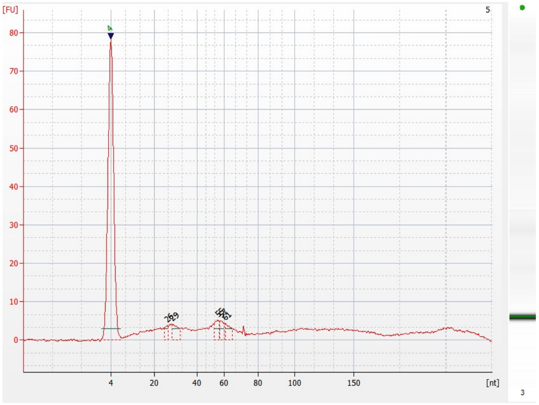

이미지 확대하기 =>2:4 - 2

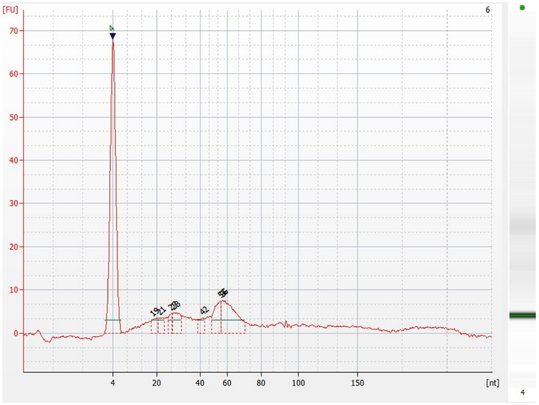

이미지 확대하기 =>3:4 - 3

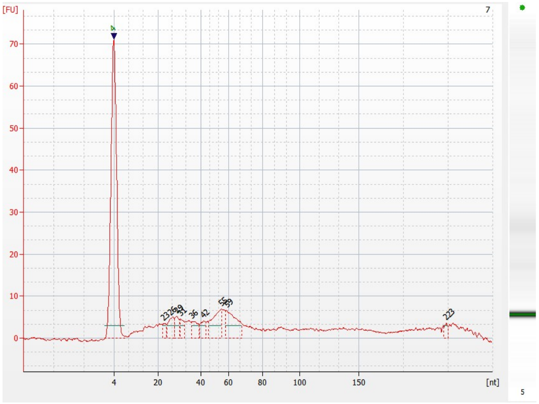

이미지 확대하기 =>4:4 - 4

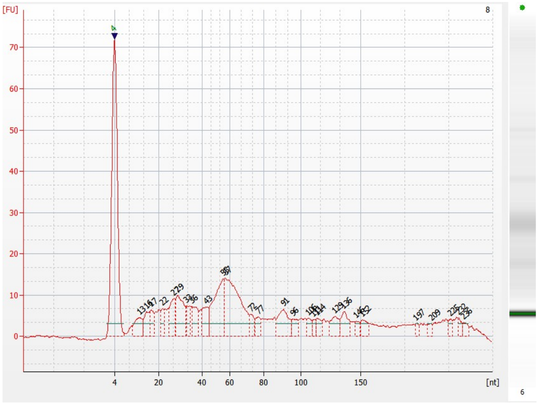

이미지 확대하기 =>5:4 - 5

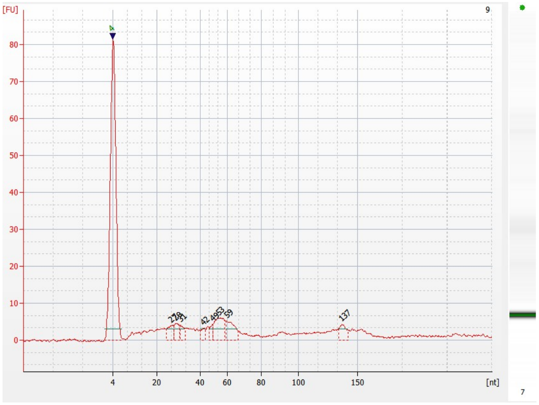

DNA QC Method

1. Quantity of DNA : Done by picogreen* method using Victor 3 fluorometry.

Macrogen quantifies the starting genomic material by a fluorescence-based quantification, rather than a UV-spectrometer-based method.
This is because fluorescence-based methods, which employ a double-stranded DNA specific dye, will specifically and accurately quantitate dsDNA even in the presence of many common contaminants. UV spectrometer methods based on 260 OD readings are prone to overestimating the DNA concentration due to the presence of RNA and other contaminants commonly found in gDNA preparations.

* Picogreen (Invitrogen, cat.#P7589)

2. Assesing the condition of the DNA : Done by gel electrophoresis method.

Gel electrophoresis is a powerful means for revealing the condition (including the presence or absence) of DNA in a sample.
Impurities, such as detergents or proteins, can be revealed by smearing of DNA bands. RNA, which interferes with 260 nm readings, is often visible at the bottom of a gel.
A ladder or smear below a band of interest may indicate nicking or other damage to DNA.

3. Size Check of DNA [Optional , Upon request, Charged]

1) DNA fragments <1kb : 2100 Bioanalyzer* is used for checking the size.

*Macrogen use DNA 1000 chip and DNA 7500chip for normal PCR product, highsensitivity chip for very small amount of DNA fragment such as ChIPed DNA.

*http://www.genomics.agilent.com/CollectionSubpage.aspx?PageType=Product&SubPageType=ProductData&PageID=1636

2) DNA fragments for PacBio and Nanopore: Femto Pulse method is used for large size of DNA fragment.

*The GQN scores the sample on a scale of 0 to 10, with 0 indicating none of the sample exceeds the threshold and 10 indicating 100 %

*Pass Criteria for size : % of over 40kb DNA > 20% (For HiFi, >30%)

RNA QC Method

1. Quality & Quantity Check of RNA : 2100 Bioanalyzer***(or 2200 TapeStation****) is used.

We check total RNA integrity using an Agilent Technologies 2100 Bioanalyzer(or 2200 TapeStation) with an RNA Integrity Number (RIN)*** value greater than or equal to 7.
RNA that has DNA contamination will result in an underestimation of the amount of RNA used. We recommends including a DNase step with the RNA isolation method.
However, contaminant DNA will be removed during mRNA purification It is very important to use high-quality RNA as the starting material.
Use of degraded RNA can result in low yield, over-representation of the 5' ends of the RNA molecules, or failure of the protocol.

***http://www.genomics.agilent.com/CollectionSubpage.aspx?PageType=Product&SubPageType=ProductData&PageID=1648

****http://www.genomics.agilent.com/article.jsp?crumbAction=push&pageId=900109

RNA QC Criteria

Platform	Library Type	Library Kit	Type	Total Amount	RIN	rRNA ratio	DV200	Viability	etc
HiSeq2500	Small RNA library	TruSeq Small RNA library	small RNA	0.100ug	-	-	-	-	
HiSeq2500	Small RNA library	TruSeq Small RNA library	exosomal RNA	0.010ug	-	-	-	-	
HiSeq2500	Small RNA library	TruSeq Small RNA library	Total RNA	3.000ug	7	1	-	-	
HiSeq2500	Small RNA library	SMARTer Small RNA library	small RNA	0.010ug	-	-	-	-	
HiSeq2500	Small RNA library	SMARTer Small RNA library	exosomal RNA	0.010ug	-	-	-	-	
HiSeq2500	Small RNA library	SMARTer Small RNA library	Total RNA	0.050ug	7	1	-	-	
HiSeq2500	Small RNA library	NEBNext Small RNA library	exosomal RNA	0.010ug	-	-	-	-	
HiSeq2500	Small RNA library	NEBNext Small RNA library	small RNA	0.100ug	-	-	-	-	
HiSeq2500	Small RNA library	NEBNext Small RNA library	Total RNA	1.000ug	7	1	-	-	
HiSeq2500	Small RNA library	QIAseq miRNA Library	Total RNA	0.500ug	7	1	-	-	
HiSeq2500	Small RNA library	QIAseq miRNA Library	small RNA	0.100ug	-	-	-	-	
HiSeq2500	Small RNA library	QIAseq miRNA Library	exosomal RNA	0.010ug	-	-	-	-	
