# Supplementary figures and images for: Potential of extracellular vesicle-derived microRNAs as a platform for biomarker discovery in acute lymphoblastic leukemia
Source: PLoS One. 2026 Jun 24;21(6):e0352501. doi: 10.1371/journal.pone.0352501 (PMC13293457; doi:10.1371/journal.pone.0352501)

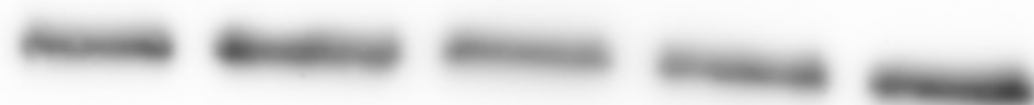

**CD9 (24 kDa)**

**CCL-119   CRL-2264   CRL-2265   CRL-3273   CRL-3274**

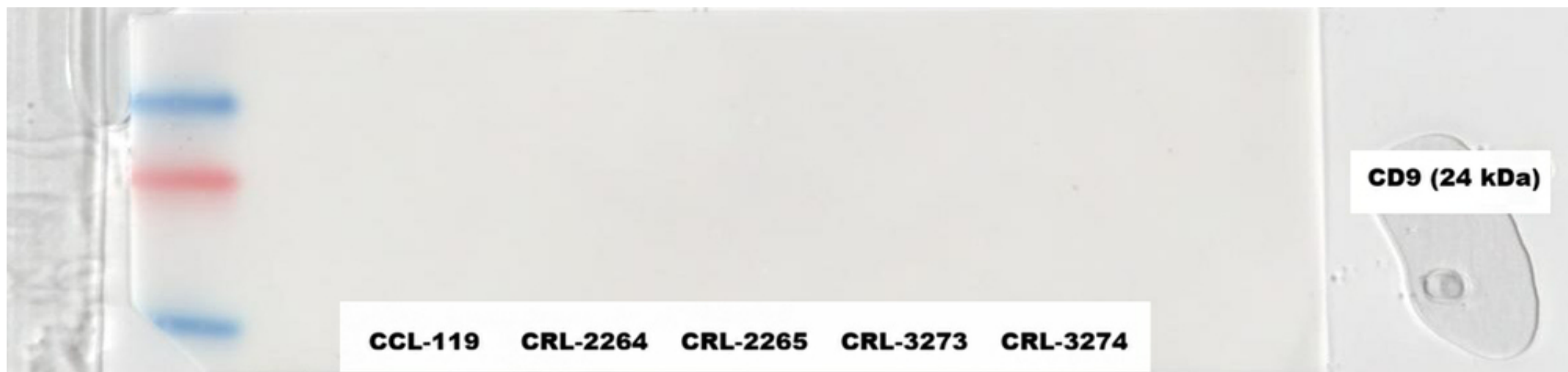

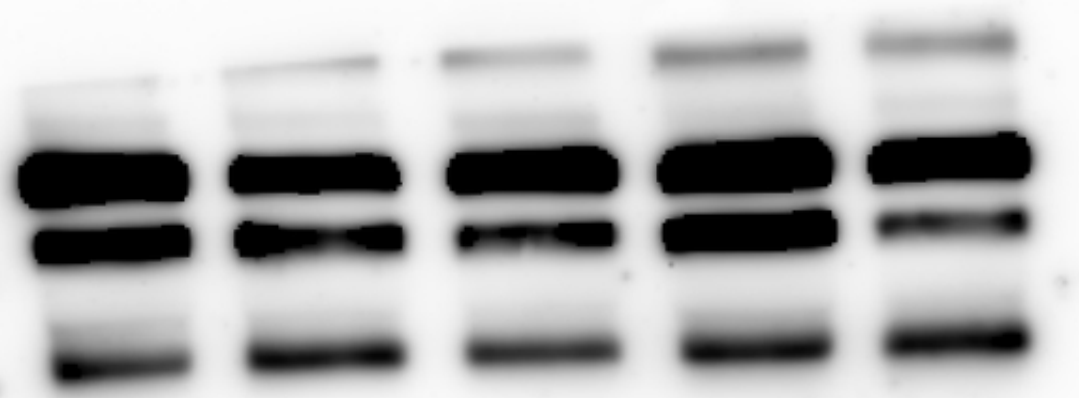

CD63 (26 kDa)

**CCL-119**

**CRL-2264**

**CRL-2265**

**CRL-3273**

**CRL-3274**

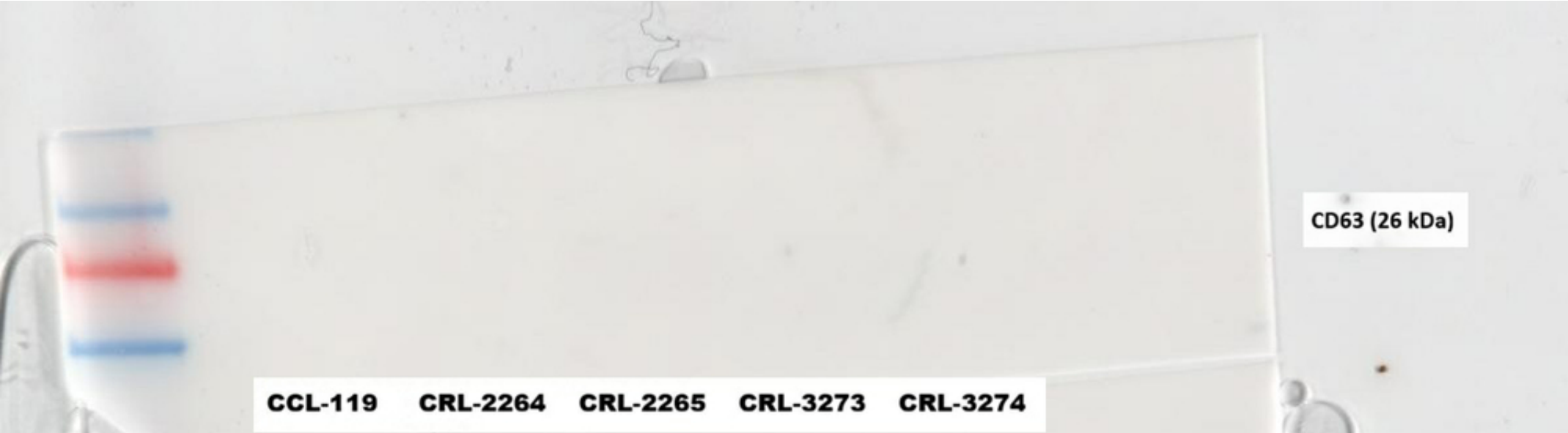

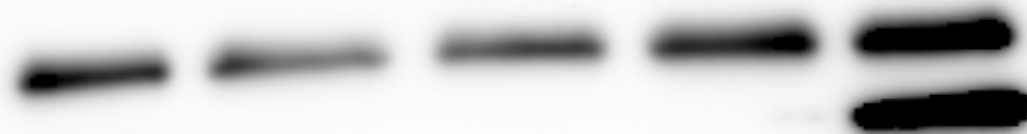

CD81 (22-26 kDa)

**CCL-119   CRL-2264   CRL-2265   CRL-3273   CRL-3274**

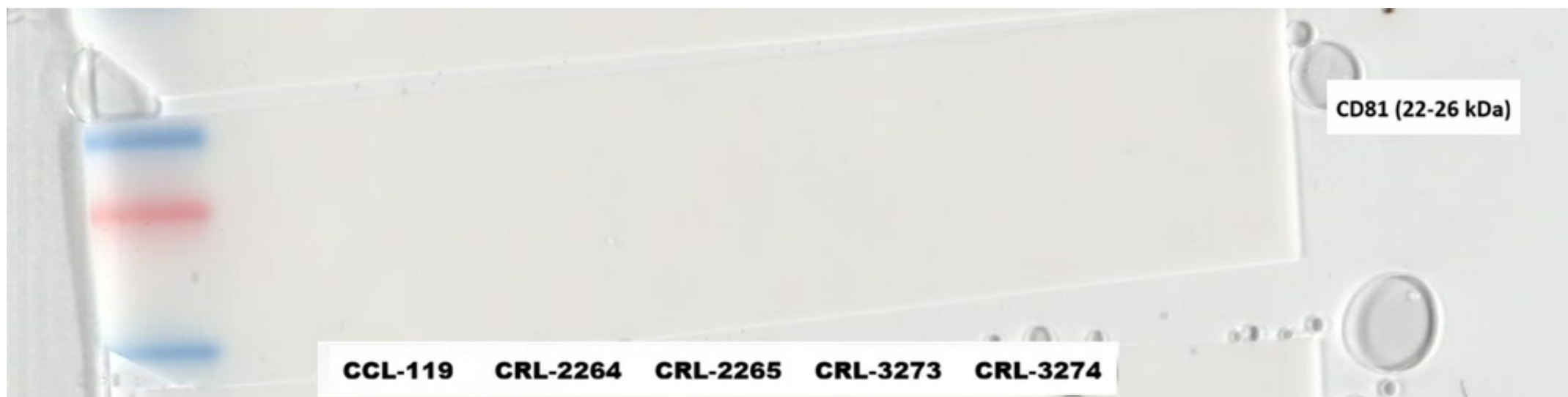

Supplement: S5 File — This file contains the raw Western blot and corresponding membrane images for EV marker proteins CD9, CD63, and CD81 in CCL-119, CRL-2264, CRL-2265, CRL-3273, and CRL-3274 samples. (PDF) [file pone.0352501.s005.pdf]
