# Supplementary material for: Potential of extracellular vesicle-derived microRNAs as a platform for biomarker discovery in acute lymphoblastic leukemia
Source: PLoS One. 2026 Jun 24;21(6):e0352501. doi: 10.1371/journal.pone.0352501 (PMC13293457; doi:10.1371/journal.pone.0352501)
Supplement: Attachment — Submitted filename: 0_Response to reviewers_20260315.docx [file pone.0352501.s008.docx]

**List of Reviewers’ comments and corresponding revisions**

**In each response, page and line numbers refer to the revised manuscript.**

**New or revised text in the manuscript is indicated with yellow highlight.**

**Journal Requirements:**

**When submitting your revision, we need you to address these additional requirements.**

**1. Please ensure that your manuscript meets PLOS ONE's style requirements, including those for file naming. The PLOS ONE style templates can be found at https://journals.plos.org/plosone/s/file?id=wjVg/PLOSOne_formatting_sample_main_body.pdf and** [**https://journals.plos.org/plosone/s/file?id=ba62/PLOSOne_formatting_sample_title_authors_affiliations.pdf**](https://journals.plos.org/plosone/s/file?id=ba62/PLOSOne_formatting_sample_title_authors_affiliations.pdf)

***Response:*** We have revised the manuscript in accordance with the style guidelines of PLOS ONE.

**2. We note that the grant information you provided in the ‘Funding Information’ and ‘Financial Disclosure’ sections do not match. When you resubmit, please ensure that you provide the correct grant numbers for the awards you received for your study in the ‘Funding Information’ section.**

***Response:*** An additional grant number has been included and described in the resubmission cover letter. The Funding Information section has also been revised accordingly.

**3. Thank you for stating the following financial disclosure:**

**“This research was conducted with the support of a 2021 ICKSH Research Project grant from the Korean Society of Hematology, which aims to discover biomarkers via the analysis of extracellular vesicles derived from chemoresistant acute lymphoblastic leukemia cell lines.”**

**Please state what role the funders took in the study. If the funders had no role, please state: "The funders had no role in study design, data collection and analysis, decision to publish, or preparation of the manuscript."**

**If this statement is not correct you must amend it as needed. Please include this amended Role of Funder statement in your cover letter; we will change the online submission form on your behalf.**

***Response:*** The disclosure has been revised as follows, and the corresponding information has also been added to the resubmission cover letter:

This work was supported by a National Research Foundation of Korea (NRF) grant funded by the Korean government (MSIT) (Grant No. NRF-2022R1G1A1006030); the ICKSH Research Project of the Korean Society of Hematology, Republic of Korea (Grant No. ICKSH-2021-01), which supports biomarker discovery through the analysis of extracellular vesicles derived from chemoresistant acute lymphoblastic leukemia cell lines; and a Korea University Grant (Grant No. K2510551). The funders had no role in the study design, data collection and analysis, decision to publish, or preparation of the manuscript.

**4. Please note that funding information should not appear in any section or other areas of your manuscript. We will only publish funding information present in the Funding Statement section of the online submission form. Please remove any funding-related text from the manuscript.**

***Response:*** The relevant content has been removed from the manuscript and is now included only in the Funding Information section.

**5. Your ethics statement should only appear in the Methods section of your manuscript. If your ethics statement is written in any section besides the Methods, please move it to the Methods section and delete it from any other section. Please ensure that your ethics statement is included in your manuscript, as the ethics statement entered into the online submission form will not be published alongside your manuscript.**

***Response:*** In accordance with the journal requirements, the Ethics Statement has been moved to the Methods section.

**6. PLOS ONE now requires that authors provide the original uncropped and unadjusted images underlying all blot or gel results reported in a submission’s figures or Supporting Information files. This policy and the journal’s other requirements for blot/gel reporting and figure preparation are described in detail at https://journals.plos.org/plosone/s/figures#loc-blot-and-gel-reporting-requirements and https://journals.plos.org/plosone/s/figures#locpreparing-figures-from-image-files. When you submit your revised manuscript, please ensure that your figures adhere fully to these guidelines and provide the original underlying images for all blot or gel data reported in your submission. See the following link for instructions on providing the original image data: https://journals.plos.org/plosone/s/figures#loc-original-images-for-blots-and-gels.**

**In your cover letter, please note whether your blot/gel image data are in Supporting Information or posted at a public data repository, provide the repository URL if relevant, and provide specific details as to which raw blot/gel images, if any, are not available. Email us at plosone@plos.org if you have any questions.**

***Response:*** The relevant content has been revised accordingly. A single PDF file containing all original blot and gel images corresponding to the main figures in the manuscript has been uploaded as Supporting Information (S1_raw_images).

**7. We notice that your supplementary figures are uploaded with the file type 'Figure'. Please amend the file type to 'Supporting Information'. Please ensure that each Supporting Information file has a legend listed in the manuscript after the references list.**

***Response:*** The relevant content has been revised accordingly.

**8. If the reviewer comments include a recommendation to cite specific previously published works, please review and evaluate these publications to determine whether they are relevant and should be cited. There is no requirement to cite these works unless the editor has indicated otherwise.**

***Response:*** The reviewers did not request the citation of any specific previously published works; therefore, no additional citations were added.

**Reviewer 1**

**Reviewer #1: In my view, this manuscript presents a relevant and timely investigation into the role of extracellular vesicle (EV)–derived microRNAs (miRNAs) as potential biomarkers for chemoresistance and prognosis in acute lymphoblastic leukemia (ALL). The authors have identified three key EV-derived miRNAs using established cell-line
models combined with bioinformatic survival analyses. The study is conceptually sound and technically well executed; however, it remains preliminary due to the lack of validation in patient-derived samples, limited statistical rigor, and incomplete functional interpretation. Addressing these aspects would substantially strengthen the manuscript’s scientific robustness and translational significance.**

**Major Points
1. Absence of Clinical Validation: The findings are limited to cell-line models. Validation using patient-derived EVs or plasma samples would be essential to confirm translational relevance and strengthen the clinical applicability of the results.**

***Response:*** We sincerely thank the reviewer for this valuable comment and agree that validation using patient-derived EVs or plasma samples would greatly strengthen the translational relevance and clinical applicability of our findings. Although this crucial point could not be addressed within the present revision, we have made every effort to revise the manuscript to appropriately reflect this limitation. In particular, we have clarified the exploratory and hypothesis-generating nature of the study, adopted more cautious wording in the Abstract and Conclusion, revised the Results section to distinguish bioinformatic exploratory analyses from direct clinical validation, and expanded the Discussion to explicitly acknowledge the absence of validation using patient-derived EV or plasma samples. We have also emphasized the need for future studies in clinically annotated patient cohorts to confirm the translational significance of these candidate biomarkers.

- To better reflect the exploratory design of the study, we revised the manuscript to use more cautious terminology and to avoid overstating the clinical interpretation of the cell line models (changing the term “chemoresistant cell lines” to “cell lines previously described as exhibiting chemoresistance-associated phenotypes”).
- We have revised the Abstract (Results section) to distinguish bioinformatic analyses from direct clinical validation and to clarify that the public dataset analysis was used solely to explore potential clinical relevance.
- We adopted more cautious wording in the Abstract (Conclusion section) to clarify that the identified miRNAs and the associated gene should be regarded as candidate biomarkers and that clinical validation in patient-derived samples remains necessary.
- We revised the study aim in the Introduction to better reflect the exploratory nature of the work and to avoid overstating the predictive or clinically validated significance of the findings (page 4, lines 21–22 and page 5, line 23).
- We revised the Results section to clarify that the GSE5314 analysis was used to explore the clinical relevance of predicted target genes and does not constitute direct validation of EV-derived miRNAs in patient samples (page 12, lines 191–192).
- We revised the Discussion to present the findings more cautiously and to distinguish the observed bioinformatic associations from direct clinical validation. We expanded the Discussion to explicitly acknowledge the absence of validation in patient-derived EVs or plasma samples as an important limitation, and we clarified that the present study should be regarded as exploratory and hypothesis-generating (page 16, lines 265–278).
- We revised the Conclusion to better align with the exploratory scope of the study and to clearly state that validation in patient-derived samples will be necessary before their clinical significance can be established (page 17, lines 302‒304).

**2. Statistical Rigor: The manuscript does not specify the number of biological replicates or apply multiple-testing corrections. To ensure data reliability, it is recommended that the authors implement false discovery rate (FDR) or comparable adjustments and clearly report replicate numbers.
*Response:*** We thank the reviewer for this important comment. We revised the Methods to clearly report information on replicates and to clarify the statistical approach. NTA measurements (particle size distribution and concentration) were performed in at three independent runs to confirm these measurements (page 6, lines 53‒56). TEM imaging and immunoblotting for CD9/CD63/CD81 were used to support EV characterization (representative results are shown). For small RNA sequencing, one representative sample per cell line was sequenced (page 7, line 86 and page 8, lines 87‒88). We performed Benjamini–Hochberg FDR adjustment; however, no miRNAs remained significant after FDR correction, given the exploratory design and limited sample size. Accordingly, nominal p-values were used for screening and ranking purposes only, and we revised the manuscript to reflect the hypothesis-generating nature of these results (page 8, lines 101‒104).

**3. Functional Interpretation: The observed upregulation of miR-29b-3p contrasts with existing studies associating this miRNA with chemosensitivity. A more detailed discussion or mechanistic explanation addressing possible context dependent effects would enhance interpretative clarity.
*Response:*** We sincerely thank the reviewer for this valuable comment and agree that the observed upregulation of EV-derived miR-29b-3p warrants a more cautious and detailed interpretation. Previous studies investigating several malignancies have linked miR-29b-3p to tumor-suppressive or chemosensitivity-related roles, but direct evidence supporting such a role in ALL remains limited. Published studies on ALL have mainly described altered expression and biological roles of miR-29b-3p, including effects on leukemia cell proliferation, apoptosis, and malignant behavior, rather than a direct role in chemotherapy response. In our study, we analyzed EV-derived miR-29b-3p rather than intracellular miR-29b-3p, and EV-associated miRNA levels may reflect selective vesicular sorting and cell-state‒dependent export rather than a uniform intracellular functional effect. Accordingly, we have revised the Discussion to clarify that the observed upregulation of EV-derived miR-29b-3p in our cell line models should be interpreted as a context-dependent, exploratory biomarker-associated observation rather than as evidence of a uniform functional role in chemoresistance. Furthermore, we added relevant references and emphasized that additional mechanistic and clinical studies are required to define the precise significance of EV-derived miR-29b-3p in ALL (page 14, lines 223‒237).

**4. Use of the GSE5314 Dataset: The rationale for employing the mRNA-based GSE5314 dataset to infer EV–miRNA related survival correlations should be clearly explained to justify its appropriateness.**

***Response:*** We thank the reviewer for this important comment and agree that the rationale for using an mRNA-based dataset should be clearly explained. GSE5314 includes gene expression profiles and survival information from patients with ALL, providing an opportunity to explore whether the predicted target genes of our EV-derived miRNAs are associated with clinically relevant survival associations. Using TargetScan, we identified *AHI1* as a high-confidence predicted target of miR-760 (context++ score > 90). Although we did not experimentally validate the miR-760–*AHI1* interaction in this study, we used this prediction to generate a hypothesis that reduced miR-760 (observed in our EV profiling of cell lines) could be linked to adverse outcomes through dysregulation of its target network. We therefore evaluated survival stratification by *AHI1* expression in GSE5314 and observed that higher *AHI1* expression was associated with poorer survival. We have revised the Methods and Discussion to clarify that this analysis was intended to provide indirect, hypothesis-generating clinical context for the predicted miRNA–mRNA axis rather than direct validation of EV-derived miRNAs in patients (page 9, lines 113‒115; and page 16, lines 270‒274).

**5. EV Characterization: The EV data presentation would benefit from including quantitative yield, purity metrics, and negative marker assessment in accordance with MISEV 2023 guidelines.
*Response:*** We thank the reviewer for this important comment. In response, we revised the manuscript to more clearly describe the EV characterization performed in this study and added a corresponding statement in the Discussion to acknowledge this limitation. Specifically, we clarified that EV characterization was based on measurements of particle size and concentration, TEM morphology, and CD9/CD63/CD81 expression, while comprehensive purity-related metrics and systematic assessment of negative markers were not included. We also revised the Discussion to indicate that the findings should be interpreted with appropriate caution in light of these limitations.

- We revised the Abstract (Methods section) to more clearly describe the EV isolation and characterization workflow, including NTA, TEM, and EV marker assessment.
- We revised the Introduction to describe the EV isolation method more cautiously and to avoid overstating the degree of purification (page 4, lines 19‒21).
- We revised the Methods to clarify that NTA was used to assess both particle size and concentration, and that western blotting was used to assess CD9, CD63, and CD81 expression (page 6, lines 49‒51 and 53‒56; and page 7, line 66).
- We revised the Results section heading to use more appropriate and cautious terminology, changing “validation” to “characterization.” We also revised the Results to explicitly state that particle concentration was quantified by NTA and that EV characterization was supported by EV morphology and marker expression.
- We expanded the Discussion to explicitly acknowledge the absence of comprehensive purity-related metrics and systematic assessment of negative markers as limitations of the present study (page 17, lines 290‒294).

**6. Depth of Discussion: The Discussion section should be expanded to integrate current evidence from recent EV–miRNA biomarker studies and highlight the broader therapeutic and prognostic implications of the findings.**

***Response:*** We thank the reviewer for this valuable comment. Accordingly, we expanded the Discussion to better integrate recent evidence from EV–miRNA biomarker studies and to place our findings in a broader translational context. Specifically, we added a discussion of recent literature highlighting that EV-associated RNAs, including miRNAs, are increasingly recognized as promising liquid biopsy biomarkers because they reflect disease biology, are protected from degradation in circulation, and may provide diagnostic and prognostic information. We also noted that EVs are not only candidate biomarker carriers but also active mediators of therapy resistance and intercellular communication, thereby supporting the potential therapeutic and prognostic relevance of EV-derived miRNAs. Finally, we revised the Discussion to emphasize that our findings should be interpreted as exploratory and hypothesis-generating, while still supporting the broader relevance of EV-derived miRNAs as candidate biomarkers in ALL (page 15, lines 251‒261 and page 16, lines 262‒264).

**Minor Points
1. The Introduction could be streamlined by summarizing epidemiological data and focusing more on the rationale for biomarker discovery.
*Response:*** We thank the reviewer for this helpful comment and have accordingly streamlined the Introduction by reducing the epidemiological and treatment-related background and placing greater emphasis on the rationale for biomarker discovery in ALL.

**2. Include RNA quality parameters, sequencing depth, and the number of biological replicates in the Methods section.
*Response:*** We thank the reviewer for this valuable comment. The Methods section has been revised to provide clearer information regarding RNA quality assessment, sequencing depth, and the number of samples included in the small RNA sequencing analysis. RNA quality and quantity were assessed using the Agilent 2100 Bioanalyzer with the RNA Pico Kit and Small RNA Kit. We also clarified that the analysis was performed using five representative ALL cell-line samples. Small RNA libraries were prepared using the SMARTer smRNA-Seq for Illumina kit and sequenced on an Illumina HiSeq 2500 platform to generate 51-bp single-end reads, with sequencing depths ranging from approximately 8.30 × 10^7^ to 9.49 × 10^7^ total reads per sample (page 7, lines 81‒86 and page 8, lines 87‒94).

**3. Add statistical annotations such as fold changes and p-values to Figures 2 and 3 for better data transparency.**

***Response:*** We thank the reviewer for this helpful suggestion. In accordance with this suggestion, we added statistical annotations (fold changes and p-values) to Figs 2 and 3 and updated the corresponding figure legends for clarity and transparency.

**4. Relocate the complete target gene list (Table 1) to supplementary material while retaining key highlights in the main text.
*Response:*** We thank the reviewer for this helpful suggestion. Accordingly, we moved the complete target gene list from Table 1 to the Supporting Information as S1 Table while retaining the key summary results in the main text (page 12, lines 186‒191).

**5. Standardize gene and miRNA nomenclature (e.g., miR-29b-3p, AHI1) and correct typographical inconsistencies.
*Response:*** We reviewed the manuscript carefully and standardized gene and miRNA nomenclature throughout, including correcting typographical inconsistencies in the text and figure legends.

**6. Provide a data repository link or accession number for RNA-seq data to enhance reproducibility.
*Response:*** We thank the reviewer for this helpful suggestion. In response, the raw small RNA-seq FASTQ files generated in this study have been deposited in the NCBI Sequence Read Archive under BioProject accession number PRJNA1433819. We have revised the Data Availability Statement accordingly to enhance reproducibility.

**7. Minor linguistic and stylistic refinements are suggested to improve overall readability and academic polish.**

***Response:*** We thank the reviewer for this helpful suggestion. In response, we carefully revised the manuscript to improve linguistic clarity, readability, and stylistic consistency. The revised manuscript was additionally edited by a professional English-language editing service (Editage) to further enhance its clarity and academic presentation.

**Reviewer #2: This study investigates EV-derived miRNAs from chemoresistant ALL cell lines to identify potential biomarkers. While the concept is interesting and clinically relevant, the manuscript has significant limitations that must be addressed:**

**1. The authors use "chemoresistant" cell lines (CRL-2264, CRL-2265, CRL-3274) but provide no experimental data demonstrating that these cells are actually resistant to chemotherapy drugs.
*Response:*** We thank the reviewer for this important comment and agree that chemoresistance was not directly validated experimentally in the present study. In the original manuscript, this terminology was based on the ATCC descriptions of the cell lines. Specifically, CRL-2264 and CRL-2265 are described as camptothecin-resistant derivatives of the parental CEM line, and CRL-3274 is described as an adriamycin-resistant variant derived from the parental NALM6 line. To avoid overstating this point, we revised the manuscript to use more cautious terminology, referring to these cells as cell lines previously described as exhibiting chemoresistance-associated phenotypes, and we clarified their relationship to the parental cell lines. We also added a corresponding limitation in the Discussion, stating that chemotherapy resistance was not directly validated experimentally within the scope of the present study (page 16, lines 280‒283).

**2. The study combines T-cell ALL (CCL-119, CRL-2264, CRL-2265) and B-cell ALL (CRL-3273, CRL-3274) with Philadelphia chromosome-positive status in the B-cell lines. These are biologically distinct entities with different miRNA landscapes and therapeutic responses. The authors acknowledge this as a limitation but don't address it
analytically.**

***Response:*** We thank the reviewer for this important comment and agree that the inclusion of both T-cell and B-cell ALL models, including Philadelphia chromosome–positive B-lineage disease, introduces biological heterogeneity. In the present study, however, our aim was not to perform a lineage-specific comparative analysis but rather to conduct an exploratory screening for EV-derived miRNAs across representative ALL cell line models, including parental and derivative lines previously described as exhibiting chemoresistance-associated phenotypes. Because conventional cytotoxic chemotherapy broadly targets rapidly proliferating cells, we considered that such an exploratory design could still be informative for identifying candidate EV-derived miRNAs associated with chemoresistance-related phenotypes. To address the reviewer’s concern, we revised the Discussion to more explicitly acknowledge this heterogeneity as an important limitation and clarified that the present findings should be interpreted cautiously as exploratory rather than subtype-specific conclusions (page 16, lines 283‒284 and page 17, lines 285‒290).

**3. While the authors perform NTA, TEM, and Western blotting for EV markers, several standard MISEV2023 guidelines are missing.
*Response:*** We thank the reviewer for this important comment. In the revised manuscript, we clarified that EVs were isolated using a commercial size-exclusion chromatography-based method and characterized by NTA (particle size distribution and concentration), TEM morphology, and immunoblotting for CD9, CD63, and CD81. We acknowledge that several additional elements emphasized in MISEV2023 (e.g., comprehensive purity-related metrics and systematic assessment of negative markers) were not incorporated, and we have explicitly added this as a limitation to ensure that the findings are interpreted with appropriate caution.
We revised the Abstract to remove overstatements regarding EV isolation purity, clarified in the Methods and Results that NTA was used to report particle concentration (page 6, lines 49‒51 and lines 53‒56; and page 10, lines 136‒138), and expanded the Discussion to acknowledge the absence of purity-related metrics and systematic assessment of negative markers as limitations (page 17, lines 290‒294).

**4. The selection of three miRNAs (miR-1226-5p, miR-760, miR-29b-3p) is based on arbitrary cutoffs (log10(RPM+1)+2 or -2) without statistical justification. The p-values reported (0.066, 0.057, 0.015) suggest only one miRNA (miR-29b-3p) meets conventional significance (p<0.05), yet all three are pursued equally.
*Response:*** We thank the reviewer for this important comment. We agree that the cutoff-based selection should be interpreted as exploratory and that the limited sample size (one small RNA-seq sample per cell line) does not support definitive statistical inference. Given this design, we prioritized miRNAs showing consistent “on/off” expression patterns between the parental and derivative lines (i.e., miRNAs absent in the parental group but present in the derivative group, or vice versa), rather than relying solely on nominal P-values. As requested, we added fold changes and p-values to Figure 2 (miR-1226-5p: P = 0.066; miR-760: P = 0.057; miR-29b-3p: P = 0.015). We also revised the text to clarify that miR-29b-3p met conventional nominal significance, whereas miR-1226-5p and miR-760 were carried forward as screening candidates based on their consistent expression patterns (page 11, lines 163‒166). Finally, we emphasize that these findings are hypothesis-generating and will require validation in patient-derived samples (page 16, lines 265‒278).

**5. The link between miR-760 and AHI1 is purely in silico (TargetScan prediction) with no experimental validation
*Response:*** We thank the reviewer for this important comment and agree that the miR-760–*AHI1* link in our study is based on in silico target prediction and has not been experimentally validated. To emphasize this, we present the miR-760–AHI1 relationship explicitly as hypothesis-generating rather than confirmatory in the revised manuscript. Specifically, we used TargetScan to identify AHI1 as a high-confidence predicted target of miR-760 (context++ score percentile = 98). We then explored the clinical relevance of the predicted target gene by survival analysis in an independent ALL cohort (GSE5314).

We have revised the text and Fig 3 legend accordingly to avoid implying direct experimental validation and to clarify that further mechanistic studies are required to confirm the miR-760–*AHI1* interaction. We also added the relevant limitations to the Discussion section (page 16, lines 265‒278).

**6. There are methodological gaps in bioinformatics analysis such as GSE5314 dataset details are vague, No multivariate survival analysis adjusting for known prognostic factors and Missing data handling not described
*Response:*** We thank the reviewer for this important comment. In response, we revised the Methods to provide additional details on the GSE5314 cohort and survival analysis workflow, including the data source, cohort size/endpoints (as available), group definition (median split), and missing-data handling. Furthermore, we clarified that the survival analysis was performed as an exploratory, univariate Kaplan–Meier analysis of predicted target genes because clinical covariates required for multivariate adjustment were unavailable (or not consistently annotated) for this dataset in the accessed portal (page 9, lines 119‒122). This point has also been explicitly acknowledged as a limitation in the revised Discussion (page 16, lines 278‒280).

**7. The study suffers from a lack of clinical translation relevance.
*Response:*** We appreciate the reviewer’s comment and agree that direct clinical validation would further strengthen the translational relevance. While patient-derived validation was not feasible within the scope of the present study, we made every effort to address this concern by clarifying the exploratory nature of the study, minimizing clinical overinterpretation, exploring clinical relevance using the GSE5314 dataset, depositing raw sequencing data to support reproducibility, and explicitly stating this limitation in the Discussion (page 16, lines 265‒278).

**8. Add statistical annotations to all graphs.
*Response:*** We thank the reviewer for this helpful suggestion. Accordingly, we added statistical annotations to all relevant graphs and updated the corresponding figure legends for clarity and transparency.

**9. Include QC metrics for RNA and sequencing data in supplementary materials.
*Response:*** We thank the reviewer for this helpful suggestion and have accordingly included RNA input QC and sequencing QC metrics in the Supporting Information (vendor QC reports), including raw read statistics (total reads, GC, Q20/Q30) and FastQC-based per-cycle quality metrics (page 8, lines 93‒94).

**10. Correct terminology throughout the manuscript.
*Response:*** We thank the reviewer for this helpful suggestion. In response, we reviewed the manuscript for terminology consistency and typographical errors, and the revised manuscript have undergone underwent professional English-language editing to further improve overall readability and clarity.

**11. The claims of the study seems overstated and the authors should tone down claims to reflect the exploratory, hypothesis-generating nature of the study and ensure conclusions match the actual data presented.**

***Response:*** We thank the reviewer for this important comment. In response, we revised the manuscript to ensure that all claims reflect the exploratory, hypothesis-generating nature of the study. Specifically, we refined the wording in the Abstract, Results, Discussion, and Conclusion; replaced definitive language with the term “candidate biomarkers”; clarified that the public dataset analysis (GSE5314) was used to explore clinical relevance rather than to provide direct clinical validation; and expanded the limitations to state that validation in patient-derived EVs or plasma samples will be required.
