# Supplementary material for: Potential of extracellular vesicle-derived microRNAs as a platform for biomarker discovery in acute lymphoblastic leukemia
Source: PLoS One. 2026 Jun 24;21(6):e0352501. doi: 10.1371/journal.pone.0352501 (PMC13293457; doi:10.1371/journal.pone.0352501)
Supplement: Attachment — Submitted filename: 0_Response to reviewers_20260427.docx [file pone.0352501.s009.docx]

**List of Reviewers’ comments and corresponding revisions**

**In each response, the page and line numbers refer to the revised manuscript.**

**New or revised text in the manuscript is indicated with yellow highlight.**

**Reviewer #1: Major Points**

**1. Lack of clinical validation (patient-derived samples) limits translational impact.**

***Response:*** We thank the reviewer for raising this important point. We agree that the absence of validation using patient-derived samples limits the translational impact of the study. In the revised manuscript, we further toned down the claims throughout the Abstract (page 2, lines 26–29, pages 2–3, lines 34–37), Introduction (pages 4–5, lines 62–63), and Conclusion (page 18, lines 353–356) and explicitly state that this is a discovery-stage, exploratory cell line study. We also emphasize that validation in patient-derived EVs or plasma samples is required before clinical interpretation.

**2. Insufficient statistical rigor (low replicates, weak significance, no robust corrections).**

***Response:*** We appreciate the reviewer’s concern regarding statistical rigor. We agree that the sequencing component is exploratory because one representative small RNA-seq library was analyzed per cell line. In the revised manuscript, we clarified the replicate structure, explicitly stated that nominal P values were used only for descriptive ranking, and avoided overinterpretation of statistical significance (page 8, lines 136–139 and page 11, lines 209–210). We also revised the figure legends and Results section to emphasize effect size and consistency across paired models rather than formal inferential testing (page 11, line 205–206 and Figure 2 legend).

**3. Functional interpretation of key miRNAs (e.g., miR-29b-3p) needs deeper mechanistic support.**

***Response:*** We thank the reviewer for this thoughtful comment. We agree that the current dataset does not provide direct mechanistic evidence for EV-derived miR-29b-3p. In the revised Discussion, we further clarified that our findings do not establish a direct mechanistic role in chemoresistance and that the observed increase may reflect context-dependent EV sorting rather than intracellular functional activity. We also expanded the discussion of prior ALL-related literature to emphasize this point. The discussion highlights that while a direct mechanism for EV-derived miR-29b-3p in chemoresistance remains unproven, these results serve as a valuable basis for future hypotheses (page 14, lines 275277 and page 15, lines 278289).

**4. Heavy reliance on in silico predictions (miR-760–AHI1) without experimental validation.**

***Response:*** We agree with the reviewer that the miR-760–*AHI1* axis is currently supported only by in silico target prediction combined with indirect clinical association analysis. In the revised manuscript, we explicitly describe this as a hypothesis-generating finding and clarify in the Methods (page 9, lines 157–161), Results (page 13, 240–241 and Figure 3 legend) and Discussion (page 16–17, lines 323–326) that direct experimental validation of miR-760–*AHI1* regulation was not performed in the present study.

**5. Biological heterogeneity (T-ALL vs B-ALL) not adequately addressed analytically.**

***Response:*** We thank the reviewer for this important point. We agree that ALL is biologically heterogeneous not only between T-ALL and B-ALL, but also within each lineage. Because our study was designed as an exploratory screen across a limited mixed cell-line panel, it was not powered for lineage-specific or subtype-specific analysis. We have therefore revised the Discussion to more clearly acknowledge this limitation, clarify the generalization of our findings, and added relevant references supporting the molecular heterogeneity of ALL and the context-dependent interpretation of EV-associated biomarkers (page 17 lines 332–342).

**6. EV characterization does not fully meet MISEV guidelines (missing purity/negative markers)**

***Response:*** We thank the reviewer for this important comment. We agree that the EV characterization does not fully satisfy the breadth of the current MISEV recommendations, particularly with respect to comprehensive purity assessment and negative-marker evaluation. The experiments were originally performed in 2022 using the characterization workflow available at that time, including SEC-based isolation, NTA, TEM, and CD9/CD63/CD81 immunoblotting. In the revised manuscript, we now state this limitation more explicitly, avoid overstating EV purity, and direct readers to the raw blot images in the Supporting Information (page 10, lines 183–184; Figure 1 legend; and page 18, lines 342–346).

**7. Methods need clearer reporting (replicates, QC metrics, sequencing depth).**

***Response:*** We agree with the reviewer’s suggestion. We have revised the Methods to clarify the replicate structure, sequencing design, and reporting framework. We also added clearer reference to the supporting QC and sequencing reports and prepared a supplementary summary table containing key QC/sequencing metrics for easier review (page 8, lines 132–133 and Supplementary Table S2). These revisions were intended to improve transparency without overloading the main text.

**8. Justification of dataset use (GSE5314) should be clearer.**

***Response:*** We thank the reviewer for this comment. We have revised the Methods (page 9, lines 157–161) and Results (page 13, lines 240–241) to clarify that GSE5314 was used only for indirect clinical contextualization at the level of predicted target genes because a publicly available ALL EV-miRNA dataset with survival information was not available. We also strengthened the Discussion to state explicitly that the GSE5314 analysis does not constitute direct validation of EV-derived miRNAs in patient samples (page 18, lines 353–356).

**9. Figures require consistent statistical annotations.**

***Response:*** We agree with the reviewer’s suggestion. We have revised the figure legends to standardize statistical reporting across the manuscript. Specifically, we clarified the meaning of error bars and run structure where relevant, distinguished nominal screening P values from the log-rank P value in the survival analysis, and made the exploratory nature of the sequencing-based candidate screening explicit in Figure 2.

**10. Improve organization (move large tables to supplementary).**

***Response:*** We appreciate this suggestion. To improve readability, we retained only the essential data presentation in the main manuscript and moved large supporting tables to the supplementary material. The full predicted target-gene list remains in the supplementary file, and additional supporting summary tables were organized there for clarity.

**11. Minor language, formatting, and nomenclature.**

***Response:*** We carefully edited the manuscript for language, formatting, and nomenclature consistency. We standardized terminology for EV-derived miRNAs, ALL lineage descriptors, parental versus derivative cell lines, and statistical wording, and we corrected minor grammatical and formatting issues throughout the manuscript**.**
