# Supplementary material for: Potential of extracellular vesicle-derived microRNAs as a platform for biomarker discovery in acute lymphoblastic leukemia
Source: PLoS One. 2026 Jun 24;21(6):e0352501. doi: 10.1371/journal.pone.0352501 (PMC13293457; doi:10.1371/journal.pone.0352501)
Supplement: Attachment — Submitted filename: 0_Response to reviewers_20260525.docx [file pone.0352501.s010.docx]

**List of Reviewers’ comments and corresponding revisions**

**In each response, the page and line numbers refer to the revised manuscript.**

**New or revised text in the manuscript is highlighted in yellow.**

**Response to reviewer #1:**

We sincerely thank the reviewer for carefully re-evaluating our revised manuscript and are grateful that the reviewer has indicated that all previous comments have been addressed. We also note the remaining concerns regarding statistical rigor, data availability, and methodological clarity. In the current round of revision, we have made additional changes to clarify the exploratory nature of the sequencing analysis, improve data availability, avoid overinterpretation of nominal statistical values, provide clearer cell line information, improve figure labeling, and expand the description of the survival analysis methodology.

**1. Statistical analysis and lack of biological replicates for sequencing**

***Response:*** We thank the reviewer for this important comment. We agree that the small RNA sequencing (RNA-seq) component lacks biological replicates and therefore cannot support replicate-based differential expression inference or formal multiple-testing-based conclusions. In the revised manuscript, we have further clarified that a single small RNA-seq library was analyzed per cell line and that replicate-based differential expression modeling and multiple-testing correction were not applied. Accordingly, the analysis was conducted as an exploratory candidate-screening analysis, with emphasis on effect size and consistent directionality across paired parental–derivative comparisons rather than formal statistical significance (Page 8, lines 137–141).

To avoid overinterpretation, we also revised the and figure legends to clarify that nominal *P* values, if shown, were used only for descriptive ranking and should not be interpreted as confirmatory statistical evidence (Figure 2 title and legend).

**2. Data availability**

***Response:*** We thank the reviewer for highlighting this issue. We agree that the underlying numerical data supporting the figures and summary results should be made more accessible. In the revised manuscript, we updated the Data Availability Statement and modified the Supporting Information files (S1–S5). The raw small RNA sequencing FASTQ files remain available in the NCBI Sequence Read Archive under BioProject accession number PRJNA1433819, as stated in the manuscript.

**3. Lack of patient validation**

***Response:*** We agree that the lack of validation using patient-derived samples is a crucial limitation. In the revised manuscript, we have further clarified that the identified extracellular vesicle (EV)-derived microRNAs (miRNAs) are candidate biomarker leads generated from cell line models and should not be interpreted as clinically validated biomarkers. We also emphasized that validation in patient-derived EVs, plasma samples, and subtype-aware clinical cohorts is required before clinical interpretation (Page 2, lines 34–35; Page 3, lines 36–37). This limitation is already discussed in the revised manuscript, where we state that direct clinical validation of the selected EV-derived miRNAs could not be performed and that the GSE5314 analysis provides only indirect, hypothesis-generating clinical context (Page 17, lines 324–328).

**4. Lack of functional validation of miRNA targets**

***Response:*** We thank the reviewer for this important point. We agree that the present study does not provide functional validation of the predicted miRNA–mRNA interactions. In the revised manuscript, we have further clarified that the miR-760–*AHI1* relationship is based on TargetScan prediction and indirect survival analysis using an mRNA dataset, and should therefore be interpreted as hypothesis-generating. Moreover, we have avoided language implying direct regulation. The revised manuscript states that the GSE5314 analysis does not directly validate EV-derived miRNAs in patients with ALL and should be interpreted as providing an indirect clinical context (Figure 3 legend and Page 17, lines 332–335).

**5. Incomplete EV characterization**

***Response:*** We thank the reviewer for this crucial comment. The EV-related experiments were performed in 2021, and the EV preparations were characterized using methods commonly used at that time, including size-exclusion chromatography-based isolation, nanoparticle tracking analysis, transmission electron microscopy, and immunoblotting for the EV-associated markers CD9, CD63, and CD81. These analyses supported the presence of EV-enriched preparations. However, we agree that the current EV reporting standards place increasing emphasis on more comprehensive characterization, including purity assessment and evaluation of depleted/negative markers. Accordingly, in the revised manuscript, we further clarified that the samples should be interpreted as EV-enriched preparations rather than fully purified EV populations (Page 10, lines 188–189). We also provided raw blot images in the Supporting Information and explicitly acknowledged this limitation in the Discussion (Page 18, lines 353–357).

**6. Clarification of B-ALL and T-ALL cell line characteristics**

***Response:*** We thank the reviewer for this valuable suggestion. To improve clarity, we added a cell line characteristics table summarizing the lineage, parental–derivative relationship, paired-comparison structure, and previously described chemoresistance-associated phenotype of each cell line (File S1 and Page 5, line 71). Furthermore, we have clarified that the mixed B-ALL/T-ALL panel was not designed or powered for lineage-specific conclusions. The revised Discussion now emphasizes that ALL heterogeneity exists both between B-ALL and T-ALL and within each lineage, and that validation in subtype-aware cohorts is required before generalization (Page 17, lines 340–347; Page 18, lines 348–351).

**7. Figure clarity and labeling**

***Response:*** We thank the reviewer for this suggestion. We revised the figures and figure legends to improve clarity, labeling, and interpretability. In Figure 1, we clarified that the data support the presence of EV-enriched preparations. In Figure 2, we clarified that the selected miRNAs represent exploratory candidates prioritized by effect size and consistent directionality rather than statistically validated differentially expressed miRNAs. In Figure 3, we clarified that the miR-760–AHI1 relationship is based on target prediction and indirect clinical contextualization rather than experimental validation.

**8. Survival analysis methodology**

***Response:*** We thank the reviewer for this helpful comment. To improve reproducibility, we have expanded the description of the survival analysis methodology. In the revised Methods section, we clarified that the GSE5314 mRNA expression cohort was used only for indirect clinical contextualization at the level of predicted target genes because publicly available ALL EV-miRNA datasets with survival information were unavailable. Furthermore, we specified that the dataset was obtained through the cBioPortal interface and analyzed using R (version 4.3.0). For each gene, samples were dichotomized into high- and low-expression groups based on the gene-specific median expression value, and samples with missing expression values were excluded from the corresponding analysis. Survival curves were estimated using the Kaplan–Meier method and compared using the log-rank test implemented in the R packages “survival” and “survminer.” We also clarified that multivariable survival analyses were not performed because the clinical covariates required for adjustment were unavailable or incompletely annotated in the accessed dataset or through the portal interface (Page 9, lines 161–172).
