# Supplementary material for: Potential of extracellular vesicle-derived microRNAs as a platform for biomarker discovery in acute lymphoblastic leukemia
Source: PLoS One. 2026 Jun 24;21(6):e0352501. doi: 10.1371/journal.pone.0352501 (PMC13293457; doi:10.1371/journal.pone.0352501)
Supplement: Peer review history

# Peer review: Decision Letter 0

**DOI:** 10.1371/journal.pone.0352501.r001

**Authors:** Francesco Bertolini

22 Feb 2026

PONE-D-25-58784

Potential of extracellular vesicle-derived microRNAs as a platform for biomarker discovery in acute lymphoblastic leukemia

PLOS One

Dear Dr. KANG,

Thank you for submitting your manuscript to PLOS ONE. After careful consideration, we feel that it has merit but does not fully meet PLOS ONE’s publication criteria as it currently stands. Therefore, we invite you to submit a revised version of the manuscript that addresses the points raised during the review process by the two Reviewers.

Please submit your revised manuscript by Apr 08 2026 11:59PM. If you will need more time than this to complete your revisions, please reply to this message or contact the journal office at plosone@plos.org. When you're ready to submit your revision, log on to https://www.editorialmanager.com/pone/ and select the 'Submissions Needing Revision' folder to locate your manuscript file.

Please include the following items when submitting your revised manuscript:

- A letter that responds to each point raised by the academic editor and reviewer(s). You should upload this letter as a separate file labeled 'Response to Reviewers'.
- A marked-up copy of your manuscript that highlights changes made to the original version. You should upload this as a separate file labeled 'Revised Manuscript with Track Changes'.
- An unmarked version of your revised paper without tracked changes. You should upload this as a separate file labeled 'Manuscript'.

If you would like to make changes to your financial disclosure, please include your updated statement in your cover letter. Guidelines for resubmitting your figure files are available below the reviewer comments at the end of this letter.

If applicable, we recommend that you deposit your laboratory protocols in protocols.io to enhance the reproducibility of your results. Protocols.io assigns your protocol its own identifier (DOI) so that it can be cited independently in the future. For instructions see: https://journals.plos.org/plosone/s/submission-guidelines#loc-laboratory-protocols. Additionally, PLOS ONE offers an option for publishing peer-reviewed Lab Protocol articles, which describe protocols hosted on protocols.io. Read more information on sharing protocols at https://plos.org/protocols?utm_medium=editorial-email&utm_source=authorletters&utm_campaign=protocols.

We look forward to receiving your revised manuscript.

Kind regards,

Francesco Bertolini, MD, PhD

Academic Editor

PLOS One

Journal Requirements:

When submitting your revision, we need you to address these additional requirements.

1.Please ensure that your manuscript meets PLOS ONE's style requirements, including those for file naming. The PLOS ONE style templates can be found at

https://journals.plos.org/plosone/s/file?id=wjVg/PLOSOne_formatting_sample_main_body.pdf and

https://journals.plos.org/plosone/s/file?id=ba62/PLOSOne_formatting_sample_title_authors_affiliations.pdf

2. We note that the grant information you provided in the ‘Funding Information’ and ‘Financial Disclosure’ sections do not match.

When you resubmit, please ensure that you provide the correct grant numbers for the awards you received for your study in the ‘Funding Information’ section.

3. Thank you for stating the following financial disclosure:

“This research was conducted with the support of a 2021 ICKSH Research Project grant from the Korean Society of Hematology, which aims to discover biomarkers via the analysis of extracellular vesicles derived from chemoresistant acute lymphoblastic leukemia cell lines.”

Please state what role the funders took in the study.  If the funders had no role, please state: "The funders had no role in study design, data collection and analysis, decision to publish, or preparation of the manuscript."

If this statement is not correct you must amend it as needed.

Please include this amended Role of Funder statement in your cover letter; we will change the online submission form on your behalf.

4. Please note that funding information should not appear in any section or other areas of your manuscript. We will only publish funding information present in the Funding Statement section of the online submission form. Please remove any funding-related text from the manuscript.

5. Your ethics statement should only appear in the Methods section of your manuscript. If your ethics statement is written in any section besides the Methods, please move it to the Methods section and delete it from any other section. Please ensure that your ethics statement is included in your manuscript, as the ethics statement entered into the online submission form will not be published alongside your manuscript.

6. PLOS ONE now requires that authors provide the original uncropped and unadjusted images underlying all blot or gel results reported in a submission’s figures or Supporting Information files. This policy and the journal’s other requirements for blot/gel reporting and figure preparation are described in detail at https://journals.plos.org/plosone/s/figures#loc-blot-and-gel-reporting-requirements and https://journals.plos.org/plosone/s/figures#loc-preparing-figures-from-image-files. When you submit your revised manuscript, please ensure that your figures adhere fully to these guidelines and provide the original underlying images for all blot or gel data reported in your submission. See the following link for instructions on providing the original image data: https://journals.plos.org/plosone/s/figures#loc-original-images-for-blots-and-gels.

In your cover letter, please note whether your blot/gel image data are in Supporting Information or posted at a public data repository, provide the repository URL if relevant, and provide specific details as to which raw blot/gel images, if any, are not available. Email us at plosone@plos.org if you have any questions.

7. We notice that your supplementary figures are uploaded with the file type 'Figure'. Please amend the file type to 'Supporting Information'. Please ensure that each Supporting Information file has a legend listed in the manuscript after the references list.

8. If the reviewer comments include a recommendation to cite specific previously published works, please review and evaluate these publications to determine whether they are relevant and should be cited. There is no requirement to cite these works unless the editor has indicated otherwise.

[Note: HTML markup is below. Please do not edit.]

Reviewer's Responses to Questions

**Comments to the Author**

1. Is the manuscript technically sound, and do the data support the conclusions?

The manuscript must describe a technically sound piece of scientific research with data that supports the conclusions. Experiments must have been conducted rigorously, with appropriate controls, replication, and sample sizes. The conclusions must be drawn appropriately based on the data presented.

Reviewer #1: Partly

Reviewer #2: Partly

**********

2. Has the statistical analysis been performed appropriately and rigorously?

Reviewer #1: No

Reviewer #2: I Don't Know

**********

3. Have the authors made all data underlying the findings in their manuscript fully available?

The PLOS Data policy requires authors to make all data underlying the findings described in their manuscript fully available without restriction, with rare exception (please refer to the Data Availability Statement in the manuscript PDF file). The data should be provided as part of the manuscript or its supporting information, or deposited to a public repository. For example, in addition to summary statistics, the data points behind means, medians and variance measures should be available. If there are restrictions on publicly sharing data—e.g. participant privacy or use of data from a third party—those must be specified.

Reviewer #1: No

Reviewer #2: Yes

**********

4. Is the manuscript presented in an intelligible fashion and written in standard English?

PLOS ONE does not copyedit accepted manuscripts, so the language in submitted articles must be clear, correct, and unambiguous. Any typographical or grammatical errors should be corrected at revision, so please note any specific errors here.

Reviewer #1: Yes

Reviewer #2: Yes

**********

5. Review Comments to the Author

Please use the space provided to explain your answers to the questions above. You may also include additional comments for the author, including concerns about dual publication, research ethics, or publication ethics. (Please upload your review as an attachment if it exceeds 20,000 characters)

Reviewer #1: In my view, this manuscript presents a relevant and timely investigation into the role of extracellular vesicle (EV)–derived microRNAs (miRNAs) as potential biomarkers for chemoresistance and prognosis in acute lymphoblastic leukemia (ALL). The authors have identified three key EV-derived miRNAs using established cell-line models combined with bioinformatic survival analyses. The study is conceptually sound and technically well executed; however, it remains preliminary due to the lack of validation in patient-derived samples, limited statistical rigor, and incomplete functional interpretation. Addressing these aspects would substantially strengthen the manuscript’s scientific robustness and translational significance.

Major Points

1.Absence of Clinical Validation: The findings are limited to cell-line models. Validation using patient-derived EVs or plasma samples would be essential to confirm translational relevance and strengthen the clinical applicability of the results.

2.Statistical Rigor: The manuscript does not specify the number of biological replicates or apply multiple-testing corrections. To ensure data reliability, it is recommended that the authors implement false discovery rate (FDR) or comparable adjustments and clearly report replicate numbers.

3.Functional Interpretation: The observed upregulation of miR-29b-3p contrasts with existing studies associating this miRNA with chemosensitivity. A more detailed discussion or mechanistic explanation addressing possible context-dependent effects would enhance interpretative clarity.

4.Use of the GSE5314 Dataset: The rationale for employing the mRNA-based GSE5314 dataset to infer EV–miRNA-related survival correlations should be clearly explained to justify its appropriateness.

5.EV Characterization: The EV data presentation would benefit from including quantitative yield, purity metrics, and negative marker assessment in accordance with MISEV 2023 guidelines.

6.Depth of Discussion: The Discussion section should be expanded to integrate current evidence from recent EV–miRNA biomarker studies and highlight the broader therapeutic and prognostic implications of the findings.

Minor Points

1.The Introduction could be streamlined by summarizing epidemiological data and focusing more on the rationale for biomarker discovery.

2.Include RNA quality parameters, sequencing depth, and the number of biological replicates in the Methods section.

3.Add statistical annotations such as fold changes and p-values to Figures 2 and 3 for better data transparency.

4.Relocate the complete target gene list (Table 1) to supplementary material while retaining key highlights in the main text.

5.Standardize gene and miRNA nomenclature (e.g., miR-29b-3p, AHI1) and correct typographical inconsistencies.

6.Provide a data repository link or accession number for RNA-seq data to enhance reproducibility.

7.Minor linguistic and stylistic refinements are suggested to improve overall readability and academic polish.

Reviewer #2: This study investigates EV-derived miRNAs from chemoresistant ALL cell lines to identify potential biomarkers. While the concept is interesting and clinically relevant, the manuscript has significant limitations that must be addressed:

1. The authors use "chemoresistant" cell lines (CRL-2264, CRL-2265, CRL-3274) but provide no experimental data demonstrating that these cells are actually resistant to chemotherapy drugs.

2. The study combines T-cell ALL (CCL-119, CRL-2264, CRL-2265) and B-cell ALL (CRL-3273, CRL-3274) with Philadelphia chromosome-positive status in the B-cell lines. These are biologically distinct entities with different miRNA landscapes and therapeutic responses. The authors acknowledge this as a limitation but don't address it analytically.

3. While the authors perform NTA, TEM, and Western blotting for EV markers, several standard MISEV2023 guidelines are missing.

4. The selection of three miRNAs (miR-1226-5p, miR-760, miR-29b-3p) is based on arbitrary cutoffs (log10(RPM+1)+2 or -2) without statistical justification. The p-values reported (0.066, 0.057, 0.015) suggest only one miRNA (miR-29b-3p) meets conventional significance (p<0.05), yet all three are pursued equally.

5. The link between miR-760 and AHI1 is purely in silico (TargetScan prediction) with no experimental validation

6. there are methodological gaps in bioinformatics analysis such as GSE5314 dataset details are vague, No multivariate survival analysis adjusting for known prognostic factors and Missing data handling not described

7. the study suffers from lack of clinical translation relevance

8. Add statistical annotations to all graphs

9. Include QC metrics for RNA and sequencing data in supplementary materials

10. Correct terminology throughout manuscript

11. the claims of the study seems overstated and the authors should tone down claims to reflect the exploratory, hypothesis-generating nature of the study and ensure conclusions match the actual data presented

**********

6. PLOS authors have the option to publish the peer review history of their article (what does this mean?). If published, this will include your full peer review and any attached files.

If you choose “no”, your identity will remain anonymous but your review may still be made public.

**Do you want your identity to be public for this peer review?**  For information about this choice, including consent withdrawal, please see our Privacy Policy.

Reviewer #1: **Yes:** DIBYASHREE CHHETRI

Reviewer #2: No

**********

[NOTE: If reviewer comments were submitted as an attachment file, they will be attached to this email and accessible via the submission site. Please log into your account, locate the manuscript record, and check for the action link "View Attachments". If this link does not appear, there are no attachment files.]

To ensure your figures meet our technical requirements, please review our figure guidelines: https://journals.plos.org/plosone/s/figures

You may also use PLOS’s free figure tool, NAAS, to help you prepare publication quality figures: https://journals.plos.org/plosone/s/figures#loc-tools-for-figure-preparation.

NAAS will assess whether your figures meet our technical requirements by comparing each figure against our figure specifications.

# Author comment: Author response to Decision Letter 1

**DOI:** 10.1371/journal.pone.0352501.r002

16 Mar 2026

List of Reviewers’ comments and corresponding revisions

In each response, page and line numbers refer to the revised manuscript.

New or revised text in the manuscript is indicated with yellow highlight.

Journal Requirements:

When submitting your revision, we need you to address these additional requirements.

1. Please ensure that your manuscript meets PLOS ONE's style requirements, including those for file naming. The PLOS ONE style templates can be found at https://journals.plos.org/plosone/s/file?id=wjVg/PLOSOne_formatting_sample_main_body.pdf and https://journals.plos.org/plosone/s/file?id=ba62/PLOSOne_formatting_sample_title_authors_affiliations.pdf

Response: We have revised the manuscript in accordance with the style guidelines of PLOS ONE.

2. We note that the grant information you provided in the ‘Funding Information’ and ‘Financial Disclosure’ sections do not match. When you resubmit, please ensure that you provide the correct grant numbers for the awards you received for your study in the ‘Funding Information’ section.

Response: An additional grant number has been included and described in the resubmission cover letter. The Funding Information section has also been revised accordingly.

3. Thank you for stating the following financial disclosure:

“This research was conducted with the support of a 2021 ICKSH Research Project grant from the Korean Society of Hematology, which aims to discover biomarkers via the analysis of extracellular vesicles derived from chemoresistant acute lymphoblastic leukemia cell lines.”

Please state what role the funders took in the study. If the funders had no role, please state: "The funders had no role in study design, data collection and analysis, decision to publish, or preparation of the manuscript."

If this statement is not correct you must amend it as needed. Please include this amended Role of Funder statement in your cover letter; we will change the online submission form on your behalf.

Response: The disclosure has been revised as follows, and the corresponding information has also been added to the resubmission cover letter:

This work was supported by a National Research Foundation of Korea (NRF) grant funded by the Korean government (MSIT) (Grant No. NRF-2022R1G1A1006030); the ICKSH Research Project of the Korean Society of Hematology, Republic of Korea (Grant No. ICKSH-2021-01), which supports biomarker discovery through the analysis of extracellular vesicles derived from chemoresistant acute lymphoblastic leukemia cell lines; and a Korea University Grant (Grant No. K2510551). The funders had no role in the study design, data collection and analysis, decision to publish, or preparation of the manuscript.

4. Please note that funding information should not appear in any section or other areas of your manuscript. We will only publish funding information present in the Funding Statement section of the online submission form. Please remove any funding-related text from the manuscript.

Response: The relevant content has been removed from the manuscript and is now included only in the Funding Information section.

5. Your ethics statement should only appear in the Methods section of your manuscript. If your ethics statement is written in any section besides the Methods, please move it to the Methods section and delete it from any other section. Please ensure that your ethics statement is included in your manuscript, as the ethics statement entered into the online submission form will not be published alongside your manuscript.

Response: In accordance with the journal requirements, the Ethics Statement has been moved to the Methods section.

6. PLOS ONE now requires that authors provide the original uncropped and unadjusted images underlying all blot or gel results reported in a submission’s figures or Supporting Information files. This policy and the journal’s other requirements for blot/gel reporting and figure preparation are described in detail at https://journals.plos.org/plosone/s/figures#loc-blot-and-gel-reporting-requirements and https://journals.plos.org/plosone/s/figures#locpreparing-figures-from-image-files. When you submit your revised manuscript, please ensure that your figures adhere fully to these guidelines and provide the original underlying images for all blot or gel data reported in your submission. See the following link for instructions on providing the original image data: https://journals.plos.org/plosone/s/figures#loc-original-images-for-blots-and-gels.

In your cover letter, please note whether your blot/gel image data are in Supporting Information or posted at a public data repository, provide the repository URL if relevant, and provide specific details as to which raw blot/gel images, if any, are not available. Email us at plosone@plos.org if you have any questions.

Response: The relevant content has been revised accordingly. A single PDF file containing all original blot and gel images corresponding to the main figures in the manuscript has been uploaded as Supporting Information (S1_raw_images).

7. We notice that your supplementary figures are uploaded with the file type 'Figure'. Please amend the file type to 'Supporting Information'. Please ensure that each Supporting Information file has a legend listed in the manuscript after the references list.

Response: The relevant content has been revised accordingly.

8. If the reviewer comments include a recommendation to cite specific previously published works, please review and evaluate these publications to determine whether they are relevant and should be cited. There is no requirement to cite these works unless the editor has indicated otherwise.

Response: The reviewers did not request the citation of any specific previously published works; therefore, no additional citations were added.

Reviewer 1

Reviewer #1: In my view, this manuscript presents a relevant and timely investigation into the role of extracellular vesicle (EV)–derived microRNAs (miRNAs) as potential biomarkers for chemoresistance and prognosis in acute lymphoblastic leukemia (ALL). The authors have identified three key EV-derived miRNAs using established cell-line

models combined with bioinformatic survival analyses. The study is conceptually sound and technically well executed; however, it remains preliminary due to the lack of validation in patient-derived samples, limited statistical rigor, and incomplete functional interpretation. Addressing these aspects would substantially strengthen the manuscript’s scientific robustness and translational significance.

Major Points

1. Absence of Clinical Validation: The findings are limited to cell-line models. Validation using patient-derived EVs or plasma samples would be essential to confirm translational relevance and strengthen the clinical applicability of the results.

Response: We sincerely thank the reviewer for this valuable comment and agree that validation using patient-derived EVs or plasma samples would greatly strengthen the translational relevance and clinical applicability of our findings. Although this crucial point could not be addressed within the present revision, we have made every effort to revise the manuscript to appropriately reflect this limitation. In particular, we have clarified the exploratory and hypothesis-generating nature of the study, adopted more cautious wording in the Abstract and Conclusion, revised the Results section to distinguish bioinformatic exploratory analyses from direct clinical validation, and expanded the Discussion to explicitly acknowledge the absence of validation using patient-derived EV or plasma samples. We have also emphasized the need for future studies in clinically annotated patient cohorts to confirm the translational significance of these candidate biomarkers.

- To better reflect the exploratory design of the study, we revised the manuscript to use more cautious terminology and to avoid overstating the clinical interpretation of the cell line models (changing the term “chemoresistant cell lines” to “cell lines previously described as exhibiting chemoresistance-associated phenotypes”).

- We have revised the Abstract (Results section) to distinguish bioinformatic analyses from direct clinical validation and to clarify that the public dataset analysis was used solely to explore potential clinical relevance.

- We adopted more cautious wording in the Abstract (Conclusion section) to clarify that the identified miRNAs and the associated gene should be regarded as candidate biomarkers and that clinical validation in patient-derived samples remains necessary.

- We revised the study aim in the Introduction to better reflect the exploratory nature of the work and to avoid overstating the predictive or clinically validated significance of the findings (page 4, lines 21–22 and page 5, line 23).

- We revised the Results section to clarify that the GSE5314 analysis was used to explore the clinical relevance of predicted target genes and does not constitute direct validation of EV-derived miRNAs in patient samples (page 12, lines 191–192).

- We revised the Discussion to present the findings more cautiously and to distinguish the observed bioinformatic associations from direct clinical validation. We expanded the Discussion to explicitly acknowledge the absence of validation in patient-derived EVs or plasma samples as an important limitation, and we clarified that the present study should be regarded as exploratory and hypothesis-generating (page 16, lines 265–278).

- We revised the Conclusion to better align with the exploratory scope of the study and to clearly state that validation in patient-derived samples will be necessary before their clinical significance can be established (page 17, lines 302‒304).

2. Statistical Rigor: The manuscript does not specify the number of biological replicates or apply multiple-testing corrections. To ensure data reliability, it is recommended that the authors implement false discovery rate (FDR) or comparable adjustments and clearly report replicate numbers.

Response: We thank the reviewer for this important comment. We revised the Methods to clearly report information on replicates and to clarify the statistical approach. NTA measurements (particle size distribution and concentration) were performed in at three independent runs to confirm these measurements (page 6, lines 53‒56). TEM imaging and immunoblotting for CD9/CD63/CD81 were used to support EV characterization (representative results are shown). For small RNA sequencing, one representative sample per cell line was sequenced (page 7, line 86 and page 8, lines 87‒88). We performed Benjamini–Hochberg FDR adjustment; however, no miRNAs remained significant after FDR correction, given the exploratory design and limited sample size. Accordingly, nominal p-values were used for screening and ranking purposes only, and we revised the manuscript to reflect the hypothesis-generating nature of these results (page 8, lines 101‒104).

3. Functional Interpretation: The observed upregulation of miR-29b-3p contrasts with existing studies associating this miRNA with chemosensitivity. A more detailed discussion or mechanistic explanation addressing possible context dependent effects would enhance interpretative clarity.

Response: We sincerely thank the reviewer for this valuable comment and agree that the observed upregulation of EV-derived miR-29b-3p warrants a more cautious and detailed interpretation. Previous studies investigating several malignancies have linked miR-29b-3p to tumor-suppressive or chemosensitivity-related roles, but direct evidence supporting such a role in ALL remains limited. Published studies on ALL have mainly described altered expression and biological roles of miR-29b-3p, including effects on leukemia cell proliferation, apoptosis, and malignant behavior, rather than a direct role in chemotherapy response. In our study, we analyzed EV-derived miR-29b-3p rather than intracellular miR-29b-3p, and EV-associated miRNA levels may reflect selective vesicular sorting and cell-state‒dependent export rather than a uniform intracellular functional effect. Accordingly, we have revised the Discussion to clarify that the observed upregulation of EV-derived miR-29b-3p in our cell line models should be interpreted as a context-dependent, exploratory biomarker-associated observation rather than as evidence of a uniform functional role in chemoresistance. Furthermore, we added relevant references and emphasized that additional mechanistic and clinical studies are required to define the precise significance of EV-derived miR-29b-3p in ALL (page 14, lines 223‒237).

4. Use of the GSE5314 Dataset: The rationale for employing the mRNA-based GSE5314 dataset to infer EV–miRNA related survival correlations should be clearly explained to justify its appropriateness.

Response: We thank the reviewer for this important comment and agree that the rationale for using an mRNA-based dataset should be clearly explained. GSE5314 includes gene expression profiles and survival information from patients with ALL, providing an opportunity to explore whether the predicted target genes of our EV-derived miRNAs are associated with clinically relevant survival associations. Using TargetScan, we identified AHI1 as a high-confidence predicted target of miR-760 (context++ score > 90). Although we did not experimentally validate the miR-760–AHI1 interaction in this study, we used this prediction to generate a hypothesis that reduced miR-760 (observed in our EV profiling of cell lines) could be linked to adverse outcomes through dysregulation of its target network. We therefore evaluated survival stratification by AHI1 expression in GSE5314 and observed that higher AHI1 expression was associated with poorer survival. We have revised the Methods and Discussion to clarify that this analysis was intended to provide indirect, hypothesis-generating clinical context for the predicted miRNA–mRNA axis rather than direct validation of EV-derived miRNAs in patients (page 9, lines 113‒115; and page 16, lines 270‒274).

5. EV Characterization: The EV data presentation would benefit from including quantitative yield, purity metrics, and negative marker assessment in accordance with MISEV 2023 guidelines.

Response: We thank the reviewer for this important comment. In response, we revised the manuscript to more clearly describe the EV characterization performed in this study and added a corresponding statement in the Discussion to acknowledge this limitation. Specifically, we clarified that EV characterization was based on measurements of particle size and concentration, TEM morphology, and CD9/CD63/CD81 expression, while comprehensive purity-related metrics and systematic assessment of negative markers were not included. We also revised the Discussion to indicate that the findings should be interpreted with appropriate caution in light of these limitations.

- We revised the Abstract (Methods section) to more clearly describe the EV isolation and characterization workflow, including NTA, TEM, and EV marker assessment.

- We revised the Introduction to describe the EV isolation method more cautiously and to avoid overstating the degree of purification (page 4, lines 19‒21).

- We revised the Methods to clarify that NTA was used to assess both particle size and concentration, and that western blotting was used to assess CD9, CD63, and CD81 expression (page 6, lines 49‒51 and 53‒56; and page 7, line 66).

- We revised the Results section heading to use more appropriate and cautious terminology, changing “validation” to “characterization.” We also revised the Results to explicitly state that particle concentration was quantified by NTA and that EV characterization was supported by EV morphology and marker expression.

- We expanded the Discussion to explicitly acknowledge the absence of comprehensive purity-related metrics and systematic assessment of negative markers as limitations of the present study (page 17, lines 290‒294).

6. Depth of Discussion: The Discussion section should be expanded to integrate current evidence from recent EV–miRNA biomarker studies and highlight the broader therapeutic and prognostic implications of the findings.

Response: We thank the reviewer for thi

Attachment Submitted filename: 0_Response to reviewers_20260315.docx

# Peer review: Decision Letter 1

**DOI:** 10.1371/journal.pone.0352501.r003

**Authors:** Francesco Bertolini, Francesco Bertolini

15 Apr 2026

PONE-D-25-58784R1

Potential of extracellular vesicle-derived microRNAs as a platform for biomarker discovery in acute lymphoblastic leukemia

PLOS One

Dear Dr. KANG,

Thank you for submitting your manuscript to PLOS ONE. After careful consideration, we feel that it has merit but does not fully meet PLOS ONE’s publication criteria as it currently stands. Therefore, we invite you to submit a revised version of the manuscript that addresses the points raised during the review process by Reviewer #1.

Please submit your revised manuscript by May 30 2026 11:59PM. If you will need more time than this to complete your revisions, please reply to this message or contact the journal office at plosone@plos.org. When you're ready to submit your revision, log on to https://www.editorialmanager.com/pone/ and select the 'Submissions Needing Revision' folder to locate your manuscript file.

Please include the following items when submitting your revised manuscript:

- A letter that responds to each point raised by the academic editor and reviewer(s). You should upload this letter as a separate file labeled 'Response to Reviewers'.
- A marked-up copy of your manuscript that highlights changes made to the original version. You should upload this as a separate file labeled 'Revised Manuscript with Track Changes'.
- An unmarked version of your revised paper without tracked changes. You should upload this as a separate file labeled 'Manuscript'.

If you would like to make changes to your financial disclosure, please include your updated statement in your cover letter. Guidelines for resubmitting your figure files are available below the reviewer comments at the end of this letter.

If applicable, we recommend that you deposit your laboratory protocols in protocols.io to enhance the reproducibility of your results. Protocols.io assigns your protocol its own identifier (DOI) so that it can be cited independently in the future. For instructions see: https://journals.plos.org/plosone/s/submission-guidelines#loc-laboratory-protocols. Additionally, PLOS ONE offers an option for publishing peer-reviewed Lab Protocol articles, which describe protocols hosted on protocols.io. Read more information on sharing protocols at https://plos.org/protocols?utm_medium=editorial-email&utm_source=authorletters&utm_campaign=protocols.

As the corresponding author, your ORCID iD is verified in the submission system and will appear in the published article. PLOS supports the use of ORCID, and we encourage all coauthors to register for an ORCID iD and use it as well. Please encourage your coauthors to verify their ORCID iD within the submission system before final acceptance, as unverified ORCID iDs will not appear in the published article. *Only*  the individual author can complete the verification step; PLOS staff *cannot*  verify ORCID iDs on behalf of authors.

We look forward to receiving your revised manuscript.

Kind regards,

Francesco Bertolini, MD, PhD

Academic Editor

PLOS One

Journal Requirements:

If the reviewer comments include a recommendation to cite specific previously published works, please review and evaluate these publications to determine whether they are relevant and should be cited. There is no requirement to cite these works unless the editor has indicated otherwise.

Please review your reference list to ensure that it is complete and correct. If you have cited papers that have been retracted, please include the rationale for doing so in the manuscript text, or remove these references and replace them with relevant current references. Any changes to the reference list should be mentioned in the rebuttal letter that accompanies your revised manuscript. If you need to cite a retracted article, indicate the article’s retracted status in the References list and also include a citation and full reference for the retraction notice.

[Note: HTML markup is below. Please do not edit.]

Reviewers' comments:

Reviewer's Responses to Questions

**Comments to the Author**

1. If the authors have adequately addressed your comments raised in a previous round of review and you feel that this manuscript is now acceptable for publication, you may indicate that here to bypass the “Comments to the Author” section, enter your conflict of interest statement in the “Confidential to Editor” section, and submit your "Accept" recommendation.

Reviewer #1: All comments have been addressed

**********

2. Is the manuscript technically sound, and do the data support the conclusions?

The manuscript must describe a technically sound piece of scientific research with data that supports the conclusions. Experiments must have been conducted rigorously, with appropriate controls, replication, and sample sizes. The conclusions must be drawn appropriately based on the data presented.

Reviewer #1: Partly

**********

3. Has the statistical analysis been performed appropriately and rigorously?

Reviewer #1: No

**********

4. Have the authors made all data underlying the findings in their manuscript fully available?

The PLOS Data policy requires authors to make all data underlying the findings described in their manuscript fully available without restriction, with rare exception (please refer to the Data Availability Statement in the manuscript PDF file). The data should be provided as part of the manuscript or its supporting information, or deposited to a public repository. For example, in addition to summary statistics, the data points behind means, medians and variance measures should be available. If there are restrictions on publicly sharing data—e.g. participant privacy or use of data from a third party—those must be specified.

Reviewer #1: Yes

**********

5. Is the manuscript presented in an intelligible fashion and written in standard English?

PLOS ONE does not copyedit accepted manuscripts, so the language in submitted articles must be clear, correct, and unambiguous. Any typographical or grammatical errors should be corrected at revision, so please note any specific errors here.

Reviewer #1: Yes

**********

6. Review Comments to the Author

Please use the space provided to explain your answers to the questions above. You may also include additional comments for the author, including concerns about dual publication, research ethics, or publication ethics. (Please upload your review as an attachment if it exceeds 20,000 characters)

Reviewer #1: Major Points

Lack of clinical validation (patient-derived samples) limits translational impact.

Insufficient statistical rigor (low replicates, weak significance, no robust corrections).

Functional interpretation of key miRNAs (e.g., miR-29b-3p) needs deeper mechanistic support.

Heavy reliance on in silico predictions (miR-760–AHI1) without experimental validation.

Biological heterogeneity (T-ALL vs B-ALL) not adequately addressed analytically.

EV characterization does not fully meet MISEV guidelines (missing purity/negative markers)

Minor Points

Methods need clearer reporting (replicates, QC metrics, sequencing depth).

Justification of dataset use (GSE5314) should be clearer.

Figures require consistent statistical annotations.

Improve organization (move large tables to supplementary).

Minor language, formatting, and nomenclature

**********

7. PLOS authors have the option to publish the peer review history of their article (what does this mean?). If published, this will include your full peer review and any attached files.

If you choose “no”, your identity will remain anonymous but your review may still be made public.

**Do you want your identity to be public for this peer review?**  For information about this choice, including consent withdrawal, please see our Privacy Policy.

Reviewer #1: No

**********

[NOTE: If reviewer comments were submitted as an attachment file, they will be attached to this email and accessible via the submission site. Please log into your account, locate the manuscript record, and check for the action link "View Attachments". If this link does not appear, there are no attachment files.]

To ensure your figures meet our technical requirements, please review our figure guidelines: https://journals.plos.org/plosone/s/figures

You may also use PLOS’s free figure tool, NAAS, to help you prepare publication quality figures: https://journals.plos.org/plosone/s/figures#loc-tools-for-figure-preparation.

NAAS will assess whether your figures meet our technical requirements by comparing each figure against our figure specifications.

# Author comment: Author response to Decision Letter 2

**DOI:** 10.1371/journal.pone.0352501.r004

27 Apr 2026

List of Reviewers’ comments and corresponding revisions

In each response, the page and line numbers refer to the revised manuscript.

New or revised text in the manuscript is indicated with yellow highlight.

Reviewer #1: Major Points

1. Lack of clinical validation (patient-derived samples) limits translational impact.

Response: We thank the reviewer for raising this important point. We agree that the absence of validation using patient-derived samples limits the translational impact of the study. In the revised manuscript, we further toned down the claims throughout the Abstract (page 2, lines 26–29, pages 2–3, lines 34–37), Introduction (pages 4–5, lines 62–63), and Conclusion (page 18, lines 353–356) and explicitly state that this is a discovery-stage, exploratory cell line study. We also emphasize that validation in patient-derived EVs or plasma samples is required before clinical interpretation.

2. Insufficient statistical rigor (low replicates, weak significance, no robust corrections).

Response: We appreciate the reviewer’s concern regarding statistical rigor. We agree that the sequencing component is exploratory because one representative small RNA-seq library was analyzed per cell line. In the revised manuscript, we clarified the replicate structure, explicitly stated that nominal P values were used only for descriptive ranking, and avoided overinterpretation of statistical significance (page 8, lines 136–139 and page 11, lines 209–210). We also revised the figure legends and Results section to emphasize effect size and consistency across paired models rather than formal inferential testing (page 11, line 205–206 and Figure 2 legend).

3. Functional interpretation of key miRNAs (e.g., miR-29b-3p) needs deeper mechanistic support.

Response: We thank the reviewer for this thoughtful comment. We agree that the current dataset does not provide direct mechanistic evidence for EV-derived miR-29b-3p. In the revised Discussion, we further clarified that our findings do not establish a direct mechanistic role in chemoresistance and that the observed increase may reflect context-dependent EV sorting rather than intracellular functional activity. We also expanded the discussion of prior ALL-related literature to emphasize this point. The discussion highlights that while a direct mechanism for EV-derived miR-29b-3p in chemoresistance remains unproven, these results serve as a valuable basis for future hypotheses (page 14, lines 275�277 and page 15, lines 278�289).

4. Heavy reliance on in silico predictions (miR-760–AHI1) without experimental validation.

Response: We agree with the reviewer that the miR-760–AHI1 axis is currently supported only by in silico target prediction combined with indirect clinical association analysis. In the revised manuscript, we explicitly describe this as a hypothesis-generating finding and clarify in the Methods (page 9, lines 157–161), Results (page 13, 240–241 and Figure 3 legend) and Discussion (page 16–17, lines 323–326) that direct experimental validation of miR-760–AHI1 regulation was not performed in the present study.

5. Biological heterogeneity (T-ALL vs B-ALL) not adequately addressed analytically.

Response: We thank the reviewer for this important point. We agree that ALL is biologically heterogeneous not only between T-ALL and B-ALL, but also within each lineage. Because our study was designed as an exploratory screen across a limited mixed cell-line panel, it was not powered for lineage-specific or subtype-specific analysis. We have therefore revised the Discussion to more clearly acknowledge this limitation, clarify the generalization of our findings, and added relevant references supporting the molecular heterogeneity of ALL and the context-dependent interpretation of EV-associated biomarkers (page 17 lines 332–342).

6. EV characterization does not fully meet MISEV guidelines (missing purity/negative markers)

Response: We thank the reviewer for this important comment. We agree that the EV characterization does not fully satisfy the breadth of the current MISEV recommendations, particularly with respect to comprehensive purity assessment and negative-marker evaluation. The experiments were originally performed in 2022 using the characterization workflow available at that time, including SEC-based isolation, NTA, TEM, and CD9/CD63/CD81 immunoblotting. In the revised manuscript, we now state this limitation more explicitly, avoid overstating EV purity, and direct readers to the raw blot images in the Supporting Information (page 10, lines 183–184; Figure 1 legend; and page 18, lines 342–346).

7. Methods need clearer reporting (replicates, QC metrics, sequencing depth).

Response: We agree with the reviewer’s suggestion. We have revised the Methods to clarify the replicate structure, sequencing design, and reporting framework. We also added clearer reference to the supporting QC and sequencing reports and prepared a supplementary summary table containing key QC/sequencing metrics for easier review (page 8, lines 132–133 and Supplementary Table S2). These revisions were intended to improve transparency without overloading the main text.

8. Justification of dataset use (GSE5314) should be clearer.

Response: We thank the reviewer for this comment. We have revised the Methods (page 9, lines 157–161) and Results (page 13, lines 240–241) to clarify that GSE5314 was used only for indirect clinical contextualization at the level of predicted target genes because a publicly available ALL EV-miRNA dataset with survival information was not available. We also strengthened the Discussion to state explicitly that the GSE5314 analysis does not constitute direct validation of EV-derived miRNAs in patient samples (page 18, lines 353–356).

9. Figures require consistent statistical annotations.

Response: We agree with the reviewer’s suggestion. We have revised the figure legends to standardize statistical reporting across the manuscript. Specifically, we clarified the meaning of error bars and run structure where relevant, distinguished nominal screening P values from the log-rank P value in the survival analysis, and made the exploratory nature of the sequencing-based candidate screening explicit in Figure 2.

10. Improve organization (move large tables to supplementary).

Response: We appreciate this suggestion. To improve readability, we retained only the essential data presentation in the main manuscript and moved large supporting tables to the supplementary material. The full predicted target-gene list remains in the supplementary file, and additional supporting summary tables were organized there for clarity.

11. Minor language, formatting, and nomenclature.

Response: We carefully edited the manuscript for language, formatting, and nomenclature consistency. We standardized terminology for EV-derived miRNAs, ALL lineage descriptors, parental versus derivative cell lines, and statistical wording, and we corrected minor grammatical and formatting issues throughout the manuscript.

Attachment Submitted filename: 0_Response to reviewers_20260427.docx

# Peer review: Decision Letter 2

**DOI:** 10.1371/journal.pone.0352501.r005

**Authors:** Francesco Bertolini, Francesco Bertolini, Francesco Bertolini

11 May 2026

PONE-D-25-58784R2

Potential of extracellular vesicle-derived microRNAs as a platform for biomarker discovery in acute lymphoblastic leukemia

PLOS One

Dear Dr. KANG,

Thank you for submitting your manuscript to PLOS ONE. After careful consideration, we feel that it has merit but does not fully meet PLOS ONE’s publication criteria as it currently stands. Therefore, we invite you to submit a revised version of the manuscript that addresses the points raised during the review process.

Please submit your revised manuscript by Jun 25 2026 11:59PM. If you will need more time than this to complete your revisions, please reply to this message or contact the journal office at plosone@plos.org. When you're ready to submit your revision, log on to https://www.editorialmanager.com/pone/ and select the 'Submissions Needing Revision' folder to locate your manuscript file.

Please include the following items when submitting your revised manuscript:

- A letter that responds to each point raised by the academic editor and reviewer(s). You should upload this letter as a separate file labeled 'Response to Reviewers'.
- A marked-up copy of your manuscript that highlights changes made to the original version. You should upload this as a separate file labeled 'Revised Manuscript with Track Changes'.
- An unmarked version of your revised paper without tracked changes. You should upload this as a separate file labeled 'Manuscript'.

If you would like to make changes to your financial disclosure, please include your updated statement in your cover letter. Guidelines for resubmitting your figure files are available below the reviewer comments at the end of this letter.

If applicable, we recommend that you deposit your laboratory protocols in protocols.io to enhance the reproducibility of your results. Protocols.io assigns your protocol its own identifier (DOI) so that it can be cited independently in the future. For instructions see: https://journals.plos.org/plosone/s/submission-guidelines#loc-laboratory-protocols. Additionally, PLOS ONE offers an option for publishing peer-reviewed Lab Protocol articles, which describe protocols hosted on protocols.io. Read more information on sharing protocols at https://plos.org/protocols?utm_medium=editorial-email&utm_source=authorletters&utm_campaign=protocols.

As the corresponding author, your ORCID iD is verified in the submission system and will appear in the published article. PLOS supports the use of ORCID, and we encourage all coauthors to register for an ORCID iD and use it as well. Please encourage your coauthors to verify their ORCID iD within the submission system before final acceptance, as unverified ORCID iDs will not appear in the published article. *Only*  the individual author can complete the verification step; PLOS staff *cannot*  verify ORCID iDs on behalf of authors.

We look forward to receiving your revised manuscript.

Kind regards,

Francesco Bertolini, MD, PhD

Academic Editor

PLOS One

Journal Requirements:

If the reviewer comments include a recommendation to cite specific previously published works, please review and evaluate these publications to determine whether they are relevant and should be cited. There is no requirement to cite these works unless the editor has indicated otherwise.

Please review your reference list to ensure that it is complete and correct. If you have cited papers that have been retracted, please include the rationale for doing so in the manuscript text, or remove these references and replace them with relevant current references. Any changes to the reference list should be mentioned in the rebuttal letter that accompanies your revised manuscript. If you need to cite a retracted article, indicate the article’s retracted status in the References list and also include a citation and full reference for the retraction notice.

[Note: HTML markup is below. Please do not edit.]

Reviewers' comments:

Reviewer's Responses to Questions

**Comments to the Author**

1. If the authors have adequately addressed your comments raised in a previous round of review and you feel that this manuscript is now acceptable for publication, you may indicate that here to bypass the “Comments to the Author” section, enter your conflict of interest statement in the “Confidential to Editor” section, and submit your "Accept" recommendation.

Reviewer #1: All comments have been addressed

**********

2. Is the manuscript technically sound, and do the data support the conclusions?

The manuscript must describe a technically sound piece of scientific research with data that supports the conclusions. Experiments must have been conducted rigorously, with appropriate controls, replication, and sample sizes. The conclusions must be drawn appropriately based on the data presented.

Reviewer #1: Yes

**********

3. Has the statistical analysis been performed appropriately and rigorously?

Reviewer #1: No

**********

4. Have the authors made all data underlying the findings in their manuscript fully available?

The PLOS Data policy requires authors to make all data underlying the findings described in their manuscript fully available without restriction, with rare exception (please refer to the Data Availability Statement in the manuscript PDF file). The data should be provided as part of the manuscript or its supporting information, or deposited to a public repository. For example, in addition to summary statistics, the data points behind means, medians and variance measures should be available. If there are restrictions on publicly sharing data—e.g. participant privacy or use of data from a third party—those must be specified.

Reviewer #1: No

**********

5. Is the manuscript presented in an intelligible fashion and written in standard English?

PLOS ONE does not copyedit accepted manuscripts, so the language in submitted articles must be clear, correct, and unambiguous. Any typographical or grammatical errors should be corrected at revision, so please note any specific errors here.

Reviewer #1: Yes

**********

6. Review Comments to the Author

Please use the space provided to explain your answers to the questions above. You may also include additional comments for the author, including concerns about dual publication, research ethics, or publication ethics. (Please upload your review as an attachment if it exceeds 20,000 characters)

Reviewer #1: The study lacks biological replicates for sequencing,patient validation,functional validation of the miRNA targets,

and complete EV characterization. Clarify B-ALL and T-ALL cell line characteristics.

Improve figure clarity and labeling.

Provide more details for survival analysis methodology.

**********

7. PLOS authors have the option to publish the peer review history of their article (what does this mean?). If published, this will include your full peer review and any attached files.

If you choose “no”, your identity will remain anonymous but your review may still be made public.

**Do you want your identity to be public for this peer review?**  For information about this choice, including consent withdrawal, please see our Privacy Policy.

Reviewer #1: **Yes:** DIBYASHREE CHHETRI

**********

[NOTE: If reviewer comments were submitted as an attachment file, they will be attached to this email and accessible via the submission site. Please log into your account, locate the manuscript record, and check for the action link "View Attachments". If this link does not appear, there are no attachment files.]

To ensure your figures meet our technical requirements, please review our figure guidelines: https://journals.plos.org/plosone/s/figures

You may also use PLOS’s free figure tool, NAAS, to help you prepare publication quality figures: https://journals.plos.org/plosone/s/figures#loc-tools-for-figure-preparation.

NAAS will assess whether your figures meet our technical requirements by comparing each figure against our figure specifications.

# Author comment: Author response to Decision Letter 3

**DOI:** 10.1371/journal.pone.0352501.r006

25 May 2026

List of Reviewers’ comments and corresponding revisions

In each response, the page and line numbers refer to the revised manuscript.

New or revised text in the manuscript is highlighted in yellow.

Response to reviewer #1:

We sincerely thank the reviewer for carefully re-evaluating our revised manuscript and are grateful that the reviewer has indicated that all previous comments have been addressed. We also note the remaining concerns regarding statistical rigor, data availability, and methodological clarity. In the current round of revision, we have made additional changes to clarify the exploratory nature of the sequencing analysis, improve data availability, avoid overinterpretation of nominal statistical values, provide clearer cell line information, improve figure labeling, and expand the description of the survival analysis methodology.

1. Statistical analysis and lack of biological replicates for sequencing

Response: We thank the reviewer for this important comment. We agree that the small RNA sequencing (RNA-seq) component lacks biological replicates and therefore cannot support replicate-based differential expression inference or formal multiple-testing-based conclusions. In the revised manuscript, we have further clarified that a single small RNA-seq library was analyzed per cell line and that replicate-based differential expression modeling and multiple-testing correction were not applied. Accordingly, the analysis was conducted as an exploratory candidate-screening analysis, with emphasis on effect size and consistent directionality across paired parental–derivative comparisons rather than formal statistical significance (Page 8, lines 137–141).

To avoid overinterpretation, we also revised the and figure legends to clarify that nominal P values, if shown, were used only for descriptive ranking and should not be interpreted as confirmatory statistical evidence (Figure 2 title and legend).

2. Data availability

Response: We thank the reviewer for highlighting this issue. We agree that the underlying numerical data supporting the figures and summary results should be made more accessible. In the revised manuscript, we updated the Data Availability Statement and modified the Supporting Information files (S1–S5). The raw small RNA sequencing FASTQ files remain available in the NCBI Sequence Read Archive under BioProject accession number PRJNA1433819, as stated in the manuscript.

3. Lack of patient validation

Response: We agree that the lack of validation using patient-derived samples is a crucial limitation. In the revised manuscript, we have further clarified that the identified extracellular vesicle (EV)-derived microRNAs (miRNAs) are candidate biomarker leads generated from cell line models and should not be interpreted as clinically validated biomarkers. We also emphasized that validation in patient-derived EVs, plasma samples, and subtype-aware clinical cohorts is required before clinical interpretation (Page 2, lines 34–35; Page 3, lines 36–37). This limitation is already discussed in the revised manuscript, where we state that direct clinical validation of the selected EV-derived miRNAs could not be performed and that the GSE5314 analysis provides only indirect, hypothesis-generating clinical context (Page 17, lines 324–328).

4. Lack of functional validation of miRNA targets

Response: We thank the reviewer for this important point. We agree that the present study does not provide functional validation of the predicted miRNA–mRNA interactions. In the revised manuscript, we have further clarified that the miR-760–AHI1 relationship is based on TargetScan prediction and indirect survival analysis using an mRNA dataset, and should therefore be interpreted as hypothesis-generating. Moreover, we have avoided language implying direct regulation. The revised manuscript states that the GSE5314 analysis does not directly validate EV-derived miRNAs in patients with ALL and should be interpreted as providing an indirect clinical context (Figure 3 legend and Page 17, lines 332–335).

5. Incomplete EV characterization

Response: We thank the reviewer for this crucial comment. The EV-related experiments were performed in 2021, and the EV preparations were characterized using methods commonly used at that time, including size-exclusion chromatography-based isolation, nanoparticle tracking analysis, transmission electron microscopy, and immunoblotting for the EV-associated markers CD9, CD63, and CD81. These analyses supported the presence of EV-enriched preparations. However, we agree that the current EV reporting standards place increasing emphasis on more comprehensive characterization, including purity assessment and evaluation of depleted/negative markers. Accordingly, in the revised manuscript, we further clarified that the samples should be interpreted as EV-enriched preparations rather than fully purified EV populations (Page 10, lines 188–189). We also provided raw blot images in the Supporting Information and explicitly acknowledged this limitation in the Discussion (Page 18, lines 353–357).

6. Clarification of B-ALL and T-ALL cell line characteristics

Response: We thank the reviewer for this valuable suggestion. To improve clarity, we added a cell line characteristics table summarizing the lineage, parental–derivative relationship, paired-comparison structure, and previously described chemoresistance-associated phenotype of each cell line (File S1 and Page 5, line 71). Furthermore, we have clarified that the mixed B-ALL/T-ALL panel was not designed or powered for lineage-specific conclusions. The revised Discussion now emphasizes that ALL heterogeneity exists both between B-ALL and T-ALL and within each lineage, and that validation in subtype-aware cohorts is required before generalization (Page 17, lines 340–347; Page 18, lines 348–351).

7. Figure clarity and labeling

Response: We thank the reviewer for this suggestion. We revised the figures and figure legends to improve clarity, labeling, and interpretability. In Figure 1, we clarified that the data support the presence of EV-enriched preparations. In Figure 2, we clarified that the selected miRNAs represent exploratory candidates prioritized by effect size and consistent directionality rather than statistically validated differentially expressed miRNAs. In Figure 3, we clarified that the miR-760–AHI1 relationship is based on target prediction and indirect clinical contextualization rather than experimental validation.

8. Survival analysis methodology

Response: We thank the reviewer for this helpful comment. To improve reproducibility, we have expanded the description of the survival analysis methodology. In the revised Methods section, we clarified that the GSE5314 mRNA expression cohort was used only for indirect clinical contextualization at the level of predicted target genes because publicly available ALL EV-miRNA datasets with survival information were unavailable. Furthermore, we specified that the dataset was obtained through the cBioPortal interface and analyzed using R (version 4.3.0). For each gene, samples were dichotomized into high- and low-expression groups based on the gene-specific median expression value, and samples with missing expression values were excluded from the corresponding analysis. Survival curves were estimated using the Kaplan–Meier method and compared using the log-rank test implemented in the R packages “survival” and “survminer.” We also clarified that multivariable survival analyses were not performed because the clinical covariates required for adjustment were unavailable or incompletely annotated in the accessed dataset or through the portal interface (Page 9, lines 161–172).

Attachment Submitted filename: 0_Response to reviewers_20260525.docx

# Peer review: Decision Letter 3

**DOI:** 10.1371/journal.pone.0352501.r007

**Authors:** Francesco Bertolini, Francesco Bertolini, Francesco Bertolini, Francesco Bertolini

11 Jun 2026

Potential of extracellular vesicle-derived microRNAs as a platform for biomarker discovery in acute lymphoblastic leukemia

PONE-D-25-58784R3

Dear Dr. KANG,

We’re pleased to inform you that your manuscript has been judged scientifically suitable for publication and will be formally accepted for publication once it meets all outstanding technical requirements.

Within one week, you’ll receive an e-mail detailing the required amendments. When these have been addressed, you’ll receive a formal acceptance letter and your manuscript will be scheduled for publication.

An invoice will be generated when your article is formally accepted. Please note, if your institution has a publishing partnership with PLOS and your article meets the relevant criteria, all or part of your publication costs will be covered. Please make sure your user information is up-to-date by logging into Editorial Manager at Editorial Manager® and clicking the ‘Update My Information' link at the top of the page. For questions related to billing, please contact billing support.

If your institution or institutions have a press office, please notify them about your upcoming paper to help maximize its impact. If they’ll be preparing press materials, please inform our press team as soon as possible -- no later than 48 hours after receiving the formal acceptance. Your manuscript will remain under strict press embargo until 2 pm Eastern Time on the date of publication. For more information, please contact onepress@plos.org.

Kind regards,

Francesco Bertolini, MD, PhD

Academic Editor

PLOS One

Additional Editor Comments (optional):

Reviewers' comments:

Reviewer's Responses to Questions

**Comments to the Author**

1. If the authors have adequately addressed your comments raised in a previous round of review and you feel that this manuscript is now acceptable for publication, you may indicate that here to bypass the “Comments to the Author” section, enter your conflict of interest statement in the “Confidential to Editor” section, and submit your "Accept" recommendation.

Reviewer #1: All comments have been addressed

**********

2. Is the manuscript technically sound, and do the data support the conclusions?

The manuscript must describe a technically sound piece of scientific research with data that supports the conclusions. Experiments must have been conducted rigorously, with appropriate controls, replication, and sample sizes. The conclusions must be drawn appropriately based on the data presented.

Reviewer #1: Yes

**********

3. Has the statistical analysis been performed appropriately and rigorously?

Reviewer #1: Yes

**********

4. Have the authors made all data underlying the findings in their manuscript fully available?

The PLOS Data policy requires authors to make all data underlying the findings described in their manuscript fully available without restriction, with rare exception (please refer to the Data Availability Statement in the manuscript PDF file). The data should be provided as part of the manuscript or its supporting information, or deposited to a public repository. For example, in addition to summary statistics, the data points behind means, medians and variance measures should be available. If there are restrictions on publicly sharing data—e.g. participant privacy or use of data from a third party—those must be specified.

Reviewer #1: Yes

**********

5. Is the manuscript presented in an intelligible fashion and written in standard English?

PLOS ONE does not copyedit accepted manuscripts, so the language in submitted articles must be clear, correct, and unambiguous. Any typographical or grammatical errors should be corrected at revision, so please note any specific errors here.

Reviewer #1: Yes

**********

6. Review Comments to the Author

Please use the space provided to explain your answers to the questions above. You may also include additional comments for the author, including concerns about dual publication, research ethics, or publication ethics. (Please upload your review as an attachment if it exceeds 20,000 characters)

Reviewer #1: The authors have satisfactorily addressed the comments raised during the review process and have significantly strengthened the manuscript. The study is presented in a clear, balanced, and scientifically rigorous manner, with appropriate acknowledgment of its limitations. The methodology and interpretation of the findings have been clarified, and the conclusions are well supported by the data and aligned with the scope of the work. I have no further concerns and recommend the manuscript for acceptance.

**********

7. PLOS authors have the option to publish the peer review history of their article (what does this mean?). If published, this will include your full peer review and any attached files.

If you choose “no”, your identity will remain anonymous but your review may still be made public.

**Do you want your identity to be public for this peer review?**  For information about this choice, including consent withdrawal, please see our Privacy Policy.

Reviewer #1: **Yes:** DIBYASHREE CHHETRI

**********

# Editor report: Acceptance letter

**DOI:** 10.1371/journal.pone.0352501.r008

**Authors:** Francesco Bertolini, Francesco Bertolini, Francesco Bertolini, Francesco Bertolini

PONE-D-25-58784R3

PLOS One

Dear Dr. KANG,

I'm pleased to inform you that your manuscript has been deemed suitable for publication in PLOS One. Congratulations! Your manuscript is now being handed over to our production team.

At this stage, our production department will prepare your paper for publication. This includes ensuring the following:

* All references, tables, and figures are properly cited

* All relevant supporting information is included in the manuscript submission,

* There are no issues that prevent the paper from being properly typeset

You will receive further instructions from the production team, including instructions on how to review your proof when it is ready. Please keep in mind that we are working through a large volume of accepted articles, so please give us a few days to review your paper and let you know the next and final steps.

Lastly, if your institution or institutions have a press office, please let them know about your upcoming paper now to help maximize its impact. If they'll be preparing press materials, please inform our press team within the next 48 hours. Your manuscript will remain under strict press embargo until 2 pm Eastern Time on the date of publication. For more information, please contact onepress@plos.org.

You will receive an invoice from PLOS for your publication fee after your manuscript has reached the completed accept phase. If you receive an email requesting payment before acceptance or for any other service, this may be a phishing scheme. Learn how to identify phishing emails and protect your accounts at https://explore.plos.org/phishing.

If we can help with anything else, please email us at customercare@plos.org.

Thank you for submitting your work to PLOS ONE and supporting open access.

Kind regards,

PLOS ONE Editorial Office Staff

on behalf of

Dr. Francesco Bertolini

Academic Editor

PLOS One
